# Fabricating Shaped and Patterned Supramolecular Multigelator Objects via Diffusion-Adhesion Gel Assembly

**DOI:** 10.1021/jacs.3c07376

**Published:** 2023-10-27

**Authors:** Chayanan Tangsombun, David K. Smith

**Affiliations:** Department of Chemistry, University of York, Heslington, York YO10 5DD, U.K.

## Abstract

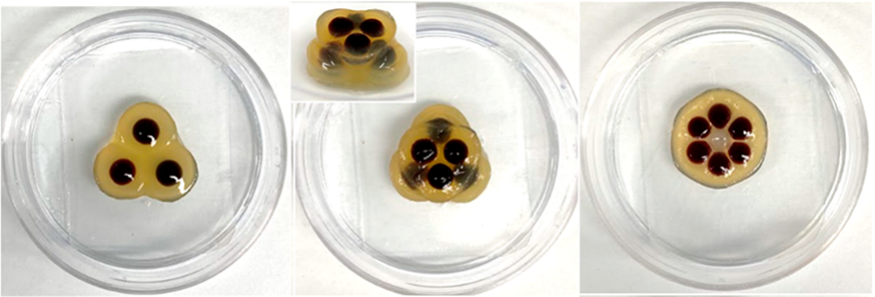

We report the use of acid-diffusion to assemble core–shell supramolecular gel beads with different low-molecular-weight gelators (LMWGs) in the core and shell. These gel beads grow a shell of dibenzylidenesorbitol-based DBS-COOH onto a core comprising DBS-CONHNH_2_ and agarose that has been loaded with acetic acid. Diffusion of the acid from the core triggers shell assembly. The presence of DBS-CONHNH_2_ enables the gel core to be loaded with metal nanoparticles (NPs) as acyl hydrazide reduces metal salts *in situ*. The pH-responsiveness of DBS-COOH allows responsive assembly of the shell with both temporal and spatial control. By fixing multiple gel beads in a Petri dish, the cores become linked to one another by the assembled DBS-COOH gel shell—a process we describe as diffusion-adhesion assembly. By controlling the geometry of the beads with respect to one another, it is possible to pattern the structures, and using a layer-by-layer approach, 3D objects can be fabricated. If some of the beads are loaded with basic DBS-carboxylate instead of CH_3_COOH, they act as a “sink” for diffusing protons, preventing DBS-COOH shell assembly in the close proximity. Those beads do not adhere to the remainder of the growing gel object and can be simply removed once diffusion-assembly is complete, acting as templates, and enabling the fabrication of 3D “imprinted” multigel architectures. Preloading the gel beads with AuNPs or AgNPs suspends these functional units within the cores at precisely defined locations within a wider gel object. In summary, this approach enables the dynamic fabrication of shaped and patterned gels with embedded metal NPs—such objects have potential next-generation applications in areas including soft nanoelectronics and regenerative medicine.

## 1 Introduction

Supramolecular gels that self-assemble from low-molecular-weight gelators (LMWGs) as a result of noncovalent interactions^1^ have wide-ranging applications from traditional industries like lubrication through to emerging areas such as nanoelectronics and regenerative medicine.^2^ Typically, such gels have relatively weak rheological properties, and break down easily under shear. Although these properties make these materials useful for some applications, they can make it challenging to shape and pattern them, which can limit their use in more sophisticated applications. In recent years, there has therefore been considerable interest in giving supramolecular gels well-defined shapes, structures, and patterns.^3^

One approach to shaping and patterning gels uses controlled reaction-diffusion. In a key study, van Esch, Eelkema and co-workers diffused solution-phase components from separate reservoirs cut into a preformed polymer gel matrix.^4^ In the locations where the two components (an acylhydrazide and an aldehyde) met, they reacted to form a self-assembling LMWG, and a gel was formed with the loading geometry controlling its shape. The same two-component gelator has also been used to glue together polymer gels—when two different gel blocks were loaded with the mutually reactive components and pushed together, the supramolecular gel formed at the interface, leading to adhesion.^5^ We have also reported a multicomponent system in which diffusion of individual components across a gel–gel interface led to interpenetrated gel networks.^6^ Others have made use of diffusion across oil–water interfaces to achieve spatially controlled gel assembly.^7^ Alternatively, the diffusion of LMWG precursors into a polymer gel loaded with an activating enzyme can generate an LMWG:PG network, and researchers have begun to explore spatial resolution.^8^

It has been recognized for some time that protonation can induce gel formation, typically by converting a carboxylate to a carboxylic acid, lowering solubility and hence triggering assembly.^9^ There has been emerging interest in using the diffusion of an acid to trigger LMWG assembly with a degree of spatial or temporal resolution. In one early study, a diffusing acid was used to assemble an LMWG, with gel fibrils being aligned with the propagating acidic diffusion wave.^10^ In important work, Besenius, Hermans and co-workers used polymer cubes presoaked in HCl and showed that a pH-responsive LMWG underwent controlled gel assembly on the surface.^11^

There has also been interest in using pH changes in enzyme-loaded polymer gels to achieve spatial and temporal control. In 2017, Jaggers and Bon used pH changes induced by urease-loaded calcium alginate gels to change the color, or induce the disassembly of gel structures.^12^ Taylor, Pojman and co-workers used this urease-driven base-releasing hydrogel to initiate a polymerization process in close proximity to the hydrogel.^13^ In an elegant study, Walther and co-workers built on this concept, demonstrating that the basic pH waves could assemble a base-responsive LMWG, and in preliminary work, began to pattern these objects.^14^

Patterning an acid catalyst on a surface can enable spatially resolved LMWG assembly via localized H+ production.^15^ Electrochemical methods have also been used to achieve spatially resolved proton release, triggering localized gel assembly.^16^ Microfluidics can also bring an acid trigger into spatially controlled contact with an LMWG.^17^ In other work, UV-irradiation of a photoacid, in combination with a mask, can create a localized source of protons within a gel, generating a gradient of acid, that subsequently diffuses through the gel, leading to a transient LMWG network assembly gradient that evolves over time.^18^

Our research group has been exploring acid diffusion as a trigger for spatially and temporally resolved gel assembly using hydrogels based on the industrially used 1,3:2,4-dibenzylidene sorbitol (DBS) scaffold.^19^ Diffusing an acid trigger through a preformed DBS-CONHNH_2_ gel matrix in a tray directed the assembly of a secondary DBS-COOH gel network with spatial resolution, creating multidomain gels that could temporally evolve in response to further changes in local pH.^20^ We went on to demonstrate that by diffusing DBS-carboxylate from one reservoir, and an acid from others, we could create well-defined gel domains where the diffusion waves overlapped, and gained a detailed understanding of the dynamics of this process.^21^

**Figure 1:**
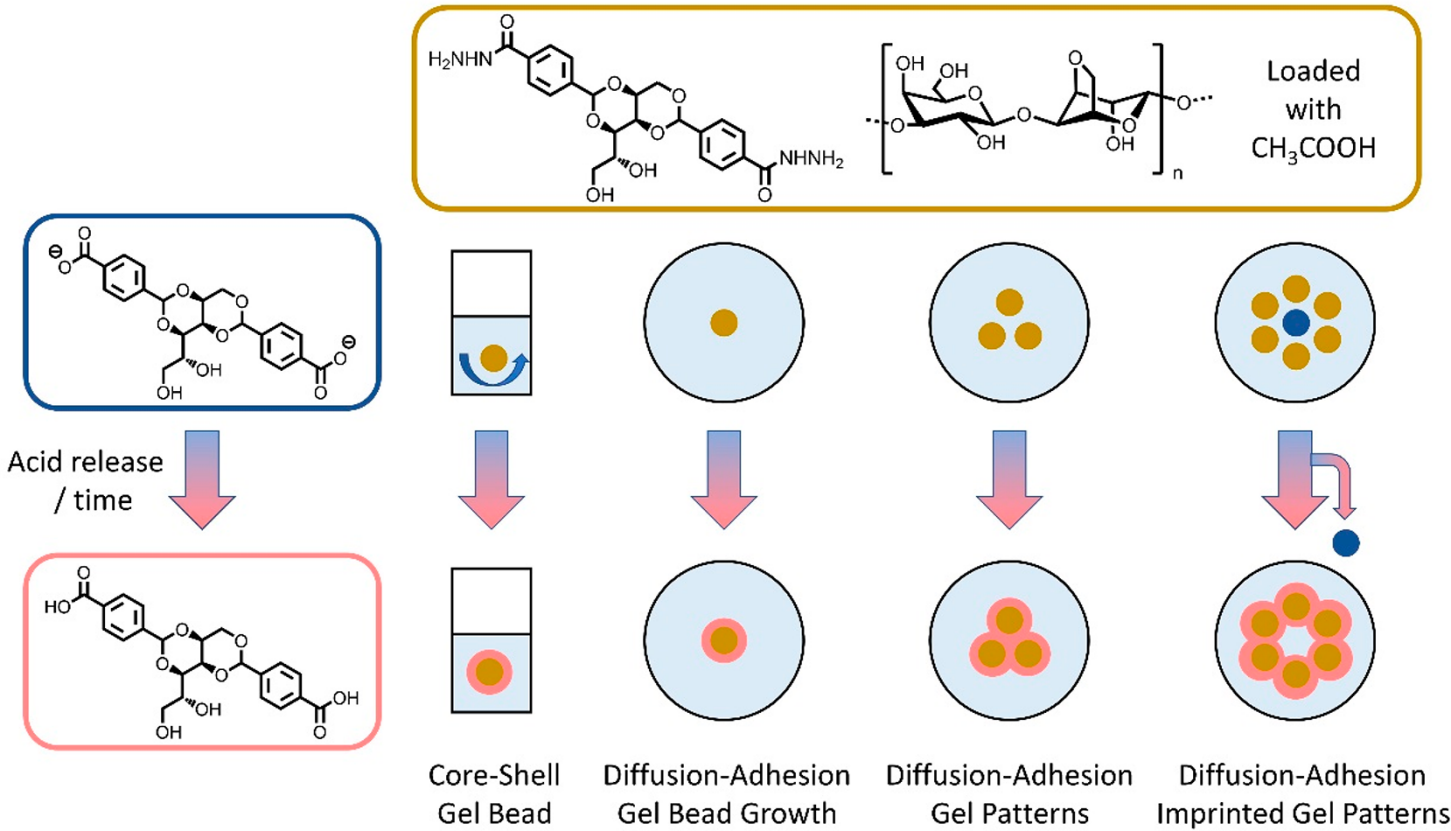
Schematic of results in this paper. Gel beads based on DBS-CONHNH_2_/Agarose are loaded with CH_3_COOH, and acid release is used to protonate proximal DBS-carboxylate, forming a self-assembled gel of DBS-COOH. This allows the formation of core–shell gel beads. By using multiple gel beads within a Petri dish, patterned core–shell gel objects can be obtained via diffusion-adhesion assembly. The presence of DBS-carboxylate loaded gel beads (shown in blue) prevents the adhesion of the DBS-COOH gel network to that bead, meaning these beads can be easily removed, acting as templates to help shape and define the remaining “imprinted” gel pattern.

In this new study, inspired by some of the landmark work on propagating pH waves,^11,14^ and wider interests in the ability of dynamic “fuelled” gel systems to generate spatially resolved systems,^22^ we wanted to develop a system in which one supramolecular gel assembled on the surface of another to generate multigelator core–shell systems (Figure 1). We reasoned that the growing shell could be used as an “adhesive” to link multiple beads together, and that this could provide access to sophisticated shaped and patterned materials—a process we describe as ‘diffusion-adhesion assembly’. Importantly, we wanted to demonstrate that both LMWGs played active roles. Shaped, dynamic, multidomain materials of this type may have future applications in soft nanoelectronics or regenerative medicine.

## 2 Results and Discussion

### 2.1 Fabrication of Core–Shell Gel Beads

Low-molecular-weight gelator (LMWG) DBS-CONHNH_2_ was synthesized using previous methods^23^ and combined with the commercial polymer gelator (PG) agarose to produce robust spherical gel beads with diameters 3.0–3.5 mm (Figure S1) using our previously disclosed approach.^24^ In brief, a hot solution of 0.3 wt %/vol LMWG and 1.0 wt %/vol PG was added dropwise (20 μL/drop) into cooled paraffin oil.

**Figure 2:**
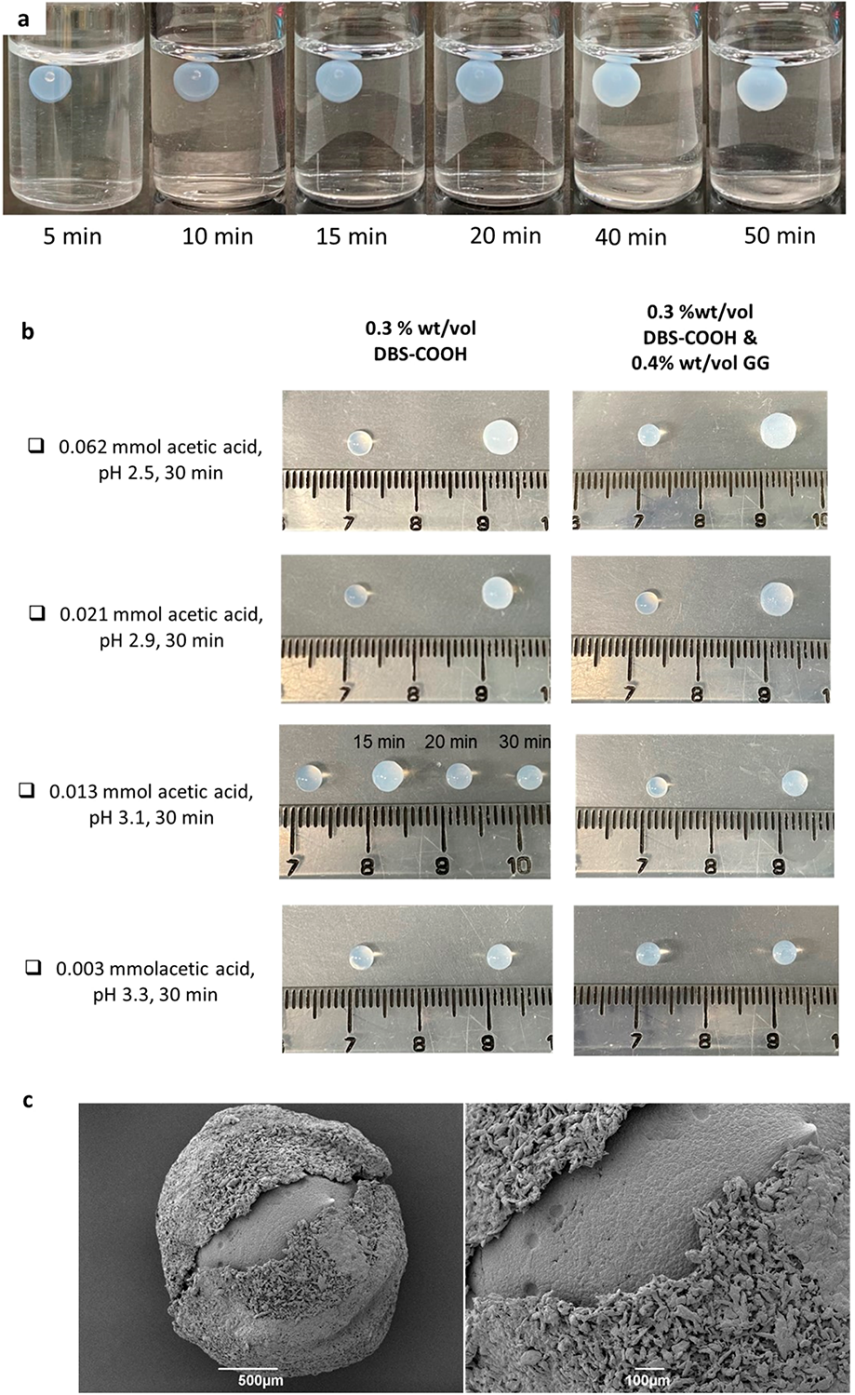
(a) Photographs of shell growth, when a DBS-CONHNH_2_/agarose gel bead loaded with citric acid (1 M) was placed in a DBS-carboxylate solution. (b) Photographs of core–shell gel bead produced by the gentle disturbance method by hand (right), compared with the initial DBS-CONHNH_2_/agarose gel bead core (left). Decreasing amounts of acetic acid (from top to bottom) give rise to smaller extents of DBS-COOH shell growth and more transient assembly. The right-hand images show the growth of the DBS-COOH shell on the core in the presence of gellan gum (GG). (c) SEM images of a DBS-CONHNH_2_/agarose gel bead with a broken DBS-COOH/GG shell. Scale bars: left 500 μm, right 100 μm.

To grow a DBS-COOH^25^ shell on the DBS-CONHNH_2_:agarose gel beads, they were loaded with an acid (fuel). Placement in a vial containing DBS-carboxylate solution enabled diffusion of the acid from the bead to induce DBS-COOH assembly on the bead surface (Figure 2a, Figure S2). After considerable optimization (see Supporting Information), we loaded CH_3_COOH (the diffusing acid) into gel beads by soaking them in solutions of different concentrations (Table S1). ^1^H NMR methods proved that DBS-CONHNH_2_ remained intact and assembled during acid-loading (Table S2, Figure S8).

Core–shell gel beads were initially fabricated by using occasional gentle disturbance of the sample vial containing the growing gel bead to prevent the bead settling on any of its surfaces. This led to reproducible spherical gel beads with visible DBS-COOH shell growth (Figure 2b). A higher loading concentration of acetic acid in the gel bead core gave rise to a larger core–shell bead, indicative of greater assembly of the DBS-COOH shell. At higher concentrations of acid, the (ca. 3.5 mm) gel bead grew to a diameter of about 5.0 mm. At lower CH_3_COOH loadings, the bead would grow for ca. 15 min, then shrink over longer time scales (Figure 2b). This suggests that on depletion of the acid, the surrounding basic solution diffuses back, neutralizing the acid wave (further discussion below). Varying the acid concentration loaded into the gel bead thus yields core–shell beads with spatiotemporal control.

Further optimization used a mechanical shaker, such that the beads remained in constant slow motion. By placing 24 well-plate arrays on the mechanical shaker, multiple core–shell gel beads could be fabricated in parallel with reproducible spherical shells (Figures S3–S4). Using this approach, the DBS-COOH shell did not grow as large as using the gentle disturbance method—e.g. at higher acid loadings, the bead grew from 3.5 mm to a diameter of ca. 4.5 mm (vs ca. 5.0 mm via gentle disturbance method). Furthermore, at lower acid concentrations, gel bead growth was not observed. Using this mechanical shaking method, the gel bead moves constantly and will therefore move more often into regions of the solution where the pH is higher (i.e., movement counteracts the pH gradient being built up by diffusion). This will somewhat limit the assembly of the DBS-COOH shell.

In general, the layer of DBS-COOH assembled on the DBS-CONHNH_2_/agarose gel bead is mechanically weak (see the rheology below). To stiffen the system, shell growth was also performed in the presence of 0.4 wt %/vol gellan gum (GG). This polymer gelator (PG) becomes incorporated into the growing DBS-COOH shell, and visibly stiffens it, making the core–shell objects more robust.^26^ Acid can also induce GG assembly^27^—we propose both DBS-CO_2_H and GG networks are activated in this way (see evidence below).

To monitor the DBS-COOH assembly, ^1^H NMR spectroscopy was used. Gelators in the liquid-like phase appear in the ^1^H NMR spectrum while gelators assembled into solid-like nanofibers are immobilized, and disappear from the spectrum.^28^ Hence, the kinetics of DBS-COOH assembly were monitored by recording NMR spectra over time in an NMR tube containing basified DBS-carboxylate solution in D_2_O (0.3 wt %/vol, pH = 12.1) with an internal standard (2 μL of DMSO), with five gel beads loaded with acetic acid (0.5 or 1.0 M) being added at time zero. For both acid concentrations, there was a rapid decrease in the DBS-COOH signal in the first 20 min and a gradual decrease after that (Figures S9–S10). The self-assembly of DBS-COOH occurs faster, and more completely, when gel beads were loaded with more concentrated acid (Figure S11), in agreement with the visualization of shell growth. DBS-COOH assembly still occurs in the presence of GG, but GG clearly has a small impact, with 10–20% less DBS-COOH being immobilized. This supports the view that GG assembly is also triggered by the diffusing acid.^27^

### 2.2 Characterization of Core–Shell Gel Beads

Having demonstrated that shells containing DBS-COOH could be grown on gel bead cores, we fabricated core–shell gel beads using the gentle disturbance method and characterized them in more detail.

IR spectroscopy (Figures S12–S13) indicated a distinctive C=O stretch in the xerogel at 1690 cm^–1^ associated with DBS-COOH assembly in the core–shell beads, supporting the hypothesis that DBS-COOH assembly takes place on the surface of the gel bead.

The thermal stability of the gels was determined by using reproducible vial inversion methodology (Table S4). For standard gels made in vials, DBS-COOH (0.3 wt %/vol) had a *T*_gel_ value at *ca*. 77 °C, similar to that of GG (*ca*. 76 °C, 0.4 wt %/vol). When DBS-COOH (0.3 wt %/vol) and GG (0.4 wt %/vol) were combined, the *T*_gel_ value increased to *ca*. 88 °C. It is reasoned that two gelators form a hybrid hydrogel with a more entangled network.^34^ Layered gels with DBS-COOH or DBS-COOH/GG as a “shell” were then also prepared in vials, using an approach that mimicked the way the beads were made (Figure S14). These “core–shell” gels underwent a two-step gel–sol transition. The DBS-COOH and DBS-COOH/GG “shells” had similar *T*_gel_ values (77 and 87 °C, respectively) to the standard DBS-COOH and DBS-COOH/GG gels described above and initially converted into sols. For the DBS-CONHNH_2_/agarose “core”, a *T*_gel_ value >100 °C was then subsequently observed, consistent with standard DBS-CONHNH_2_/agarose gel as a result of the presence of agarose.

Rheological measurements using a parallel plate geometry were performed on gels made in vials (Table 1, Figures S15–S21). The *G*′ value of the DBS-COOH and GG combination was *ca*. 5300 Pa, which was significantly stiffer than individual DBS-COOH (*G*′ = 400 Pa) or GG (*G*′ = *ca*. 470 Pa), supporting the view that the two gelators form interpenetrating networks on acid diffusion, enhancing mechanical performance. The DBS-CONHNH_2_/agarose hybrid gels were also stiff, with *G*′ values of *ca*. 13500 Pa, demonstrating their suitability for use as gel bead cores. The “core–shell” two-layer gels were less stiff than the DBS-CONHNH_2_/agarose lower layer on its own, but it was surprising that they did not lose more of their rheological stability. The system with a second layer of DBS-COOH alone was softer (*G*′ = 7625 Pa) than that with a hybrid DBS-COOH/GG upper layer (*G*′ = 10300 Pa), again demonstrating the mechanical benefits of the supporting PG network in the “shell”.

**Table 1:** Rheological Performance of Gels Formed in Vials: *G*′ (Elastic Modulus), *G*″ (Viscous Modulus), and % Shear Strain at Which *G*′ = *G*′′^a^

Gel	*G*′ (Pa)	*G*′′ (Pa)	% Shear strain at *G*′ = *G*′′
DBS-COOH	400	70	25.2
GG	475	75	2.5
DBS-COOH/GG	5315	350	5.8
DBS-CONHNH_2_/Agarose	13 460	940	3.5
DBS-COOH on DBS-CONHNH_2_/Agarose	7625	470	6.2
DBS-COOH/GG on DBS-CONHNH_2_/Agarose	10 300	550	5.7

a For samples in which one gel system is described as being “on” another, the gel was made as two layers in the vial in a way that mimicked the formation of core-shell gel beads–i.e. by diffusion of acetic acid from the lower layer to trigger assembly of the upper layer.

Scanning electron microscopy (SEM) was used to visualize the nanoscale structure and morphology. The surface of the gel bead was densely packed, and the DBS-CONHNH_2_/agarose core had a nanostructured fibrillar interior (Figures S22–S24). The DBS-COOH hydrogel shell was delicate, and it was not possible to obtain a clean cut. When the surface shell had been further stiffened by the presence of GG, the core–shell gel bead revealed a rough surface (Figure S25), and in one informative case there was evidence of the interface between core and shell due to the shell being partly broken (Figure 2c and Figures S26–S28). This breakage, which was not observed for any of the beads with DBS-COOH shells alone, presumably reflects the greater stiffness of the DBS-COOH/GG shell. The surface of the core had a uniform wrinkled texture, in agreement with previous reports of such gel beads,^24^ while the shell exhibited a sponge-like DBS-COOH network with densely packed GG.

### 2.3 Growth from Immobilized Gel Beads to Create Multidomain Gel Objects via Diffusion-Adhesion

**Figure 3:**
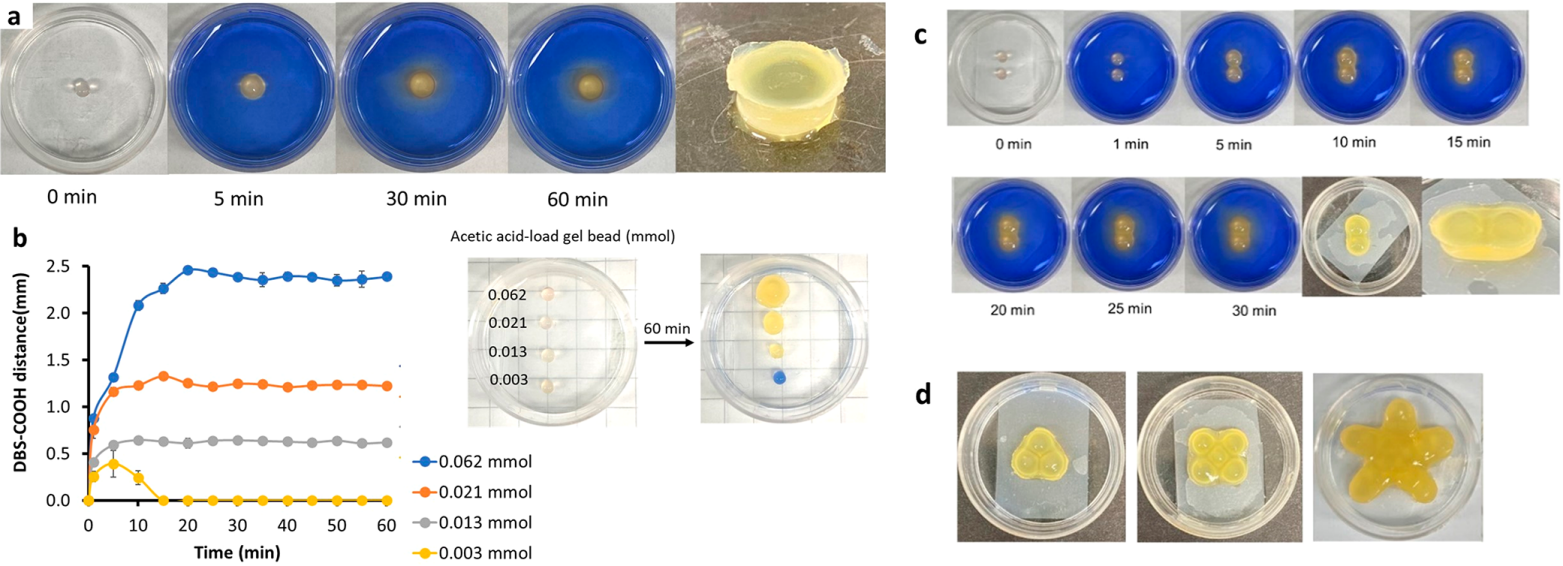
(a) Photographs of a DBS-COOH gel over time after placement of one 0.062 mmol acetic acid-loaded gel bead in DBS-carboxylate solution (0.3 wt %/vol, 3 mL) in a 3.5 cm Petri dish. (b) The distance of CH_3_COOH diffusion as determined by the assembly radius of the DBS-COOH shell into an opaque gel over time at different concentrations, and photographs indicating the growth of the gel beads, and indicated pH values after 60 min. (c) The formation of free-standing hydrogel objects from two acetic acid-loaded gel beads (0.062 mmol/bead) in a 3.5 cm Petri dish containing DBS-carboxylate (0.3 wt %/vol, 3 mL), as a result of DBS-COOH assembly via diffusion-adhesion. (d) The formation of triangle, square and star-shaped hydrogels from gel beads loaded with acetic acid (0.062 mmol/bead) in a 3.5 cm Petri dish containing DBS-carboxylate (0.3 wt %/vol, 3 mL).

Having generated symmetrical core–shell gel beads, as described above, we wanted to use this strategy to create more complex objects. We reasoned that gel beads could be placed in a Petri dish, giving them fixed locations, and allowed to grow as a result of shell formation. This opens the possibility of growing objects, potentially using multiple gel beads, with geometric control, to create unique shaped gels with multiple core–shell objects embedded in them (Figure 3).

To visualize these experiments, we used a pH indicator—thymol blue. Gel beads of the same size were immersed in different concentrations of CH_3_COOH (1.00, 0.50, 0.25, or 0.10 M). Acid-loaded gel beads were indicated as yellow after loading for 30 min. The acid-loaded gel beads were then placed in a Petri dish (3.5 cm diameter), and basic DBS-carboxylate solution (0.3 wt %/vol 3 mL, with thymol blue) was loaded. In each case, CH_3_COOH diffused from the gel beads into the DBS-carboxylate solution, as indicated by the color change from blue to yellow (Figure 3a). During the diffusion process, the DBS-carboxylate was protonated and DBS-COOH assembled into a solid-like network, resulting in the formation of an opaque gel domain around the gel bead. The solution height was selected to match the diameter of the gel bead, limiting growth from the gel bead into “two dimensions”. This led to a roughly cylindrical object constrained by the Petri dish and the air–water interface at the bottom and top, respectively. Considering rates of growth over the first minute, the shell grew at 14.7, 12.5, 6.8, and 4.3 μm/s based on CH_3_COOH loadings of 62, 21, 13, and 3 μmol, respectively. These gel growth rates are slightly faster than those previously reported by Besenius, Hermans and co-workers for diffusion-driven gel assembly (ca. 3 μm/s).^11^

The growth of the DBS-COOH shell was controlled by the amount of acid loaded into the gel bead, with more acid giving a larger DBS-COOH assembly zone (Figure 3b, Figure S29). For gel beads with ≥0.25 M acetic acid loading, the DBS-COOH network grows and then remains stable for ca. 20 min. In contrast, for gel beads loaded with only 0.10 M acetic acid, growth of a DBS-COOH shell was then followed by disassembly as the surrounding basic solution diffuses back, neutralizing the acid wave, as shown by the blue color in the gel bead after 60 min (Figure 3b). Thus, varying the acid concentration can provide transient objects with spatiotemporal control, the same as what had been observed for the core–shell gel beads described above. Unlike many approaches to gel shaping, therefore, this process is based on a dynamic process which can yield objects that are either temporally stable or transient in nature.

We then applied this process, using multiple beads in close proximity, to create more complex materials. This simple method constitutes an innovative and dynamic “top-down” approach to gel shaping and patterning. Shaping relied on the spatial positioning of acid-loaded gel beads, with acid diffusion leading to adhesion between the beads, mediated by the assembly of an interpenetrated DBS-COOH shell—a process we refer to as diffusion-adhesion. Initially, two gel beads loaded with acetic acid (1 M) were arranged on parafilm and immersed in basic DBS-carboxylate solution (0.3 wt %/vol, 3 mL) in a Petri dish (Figure 3c). If the beads moved, they were gently rearranged using a spatula before diffusion started. After a few minutes, the acid had diffused in all directions from each gel bead. The acid diffusion wave emanating from one gel bead overlapped with that from the other to form an oval-shaped hydrogel “bridge” after ca. 5 min. In this way, adhesion between the two gel beads was achieved, mediated by the growing shell of DBS-COOH as a consequence of proton diffusion. When the remaining DBS-carboxylate solution was removed, a self-standing shaped hydrogel was obtained, with the pattern being programmed by the initial geometry of the gel bead array combined with diffusion dynamics. Clearly, the kinetics of DBS-COOH assembly are fast in this system, allowing growth of the adhesive shell in this way. Given the physical manipulation, we suggest this approach has a resolution limit of ca. 1 mm bead size and 1 mm spacing between beads.

By arranging beads in different starting geometries, shapes such as triangular, square, or star patterns were obtained (Figure 3d, Figures S30–S32). By visual observation of the indicator color change, it was evident that the pH fell more quickly as more acid diffused from the larger number of gel beads. In each case, self-standing objects could be removed from the solution, demonstrating the effectiveness of adhesion between the individual beads.

**Figure 4:**
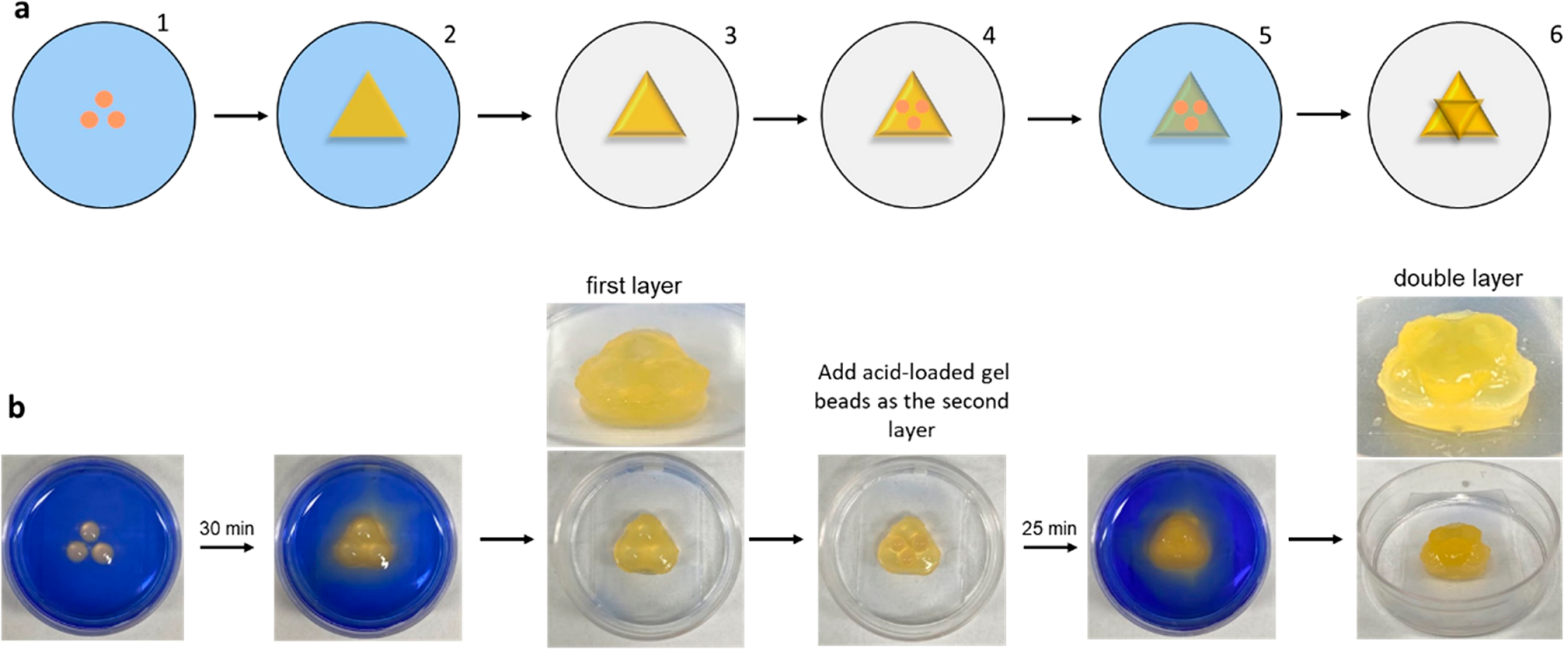
(a) Schematic diagram showing a double triangle fabricated by a layer-by-layer approach: (1) CH_*3*_COOH-loaded gel beads (0.062 mmol/bead) are placed in a 3.5 cm Petri dish containing DBS-carboxylate. (2) The acid diffuses to induce DBS-COOH gel assembly, producing a triangle. (3) DBS-carboxylate is removed to yield a free-standing object. (4) Three CH_3_COOH-loaded gel beads (0.062 mmol/bead) are arranged in an inverted triangle on top of the triangular gel. (5) The pattern is immersed in DBS-carboxylate solution, with diffusion adhering the beads in the upper triangle both to each other and the lower triangle. (6) Removal of DBS-carboxylate furnishes the double triangle. (b) Photographs illustrating the schematic approach.

A more complex double-layer structure was then developed from the triangle pattern by the addition of a second layer of acid-loaded gel beads after the first layer of beads had been allowed to adhere (Figure 4). In addition to the three upper gel beads adhering to one another, they also become attached to the lower layer as the acid diffuses in all directions from the gel bead. This demonstrates how 3D architectures can readily be achieved by using this diffusion-adhesion approach.

### 2.4 Imprinting in Diffusion-Adhesion Gel Bead Assembly

**Figure 5:**
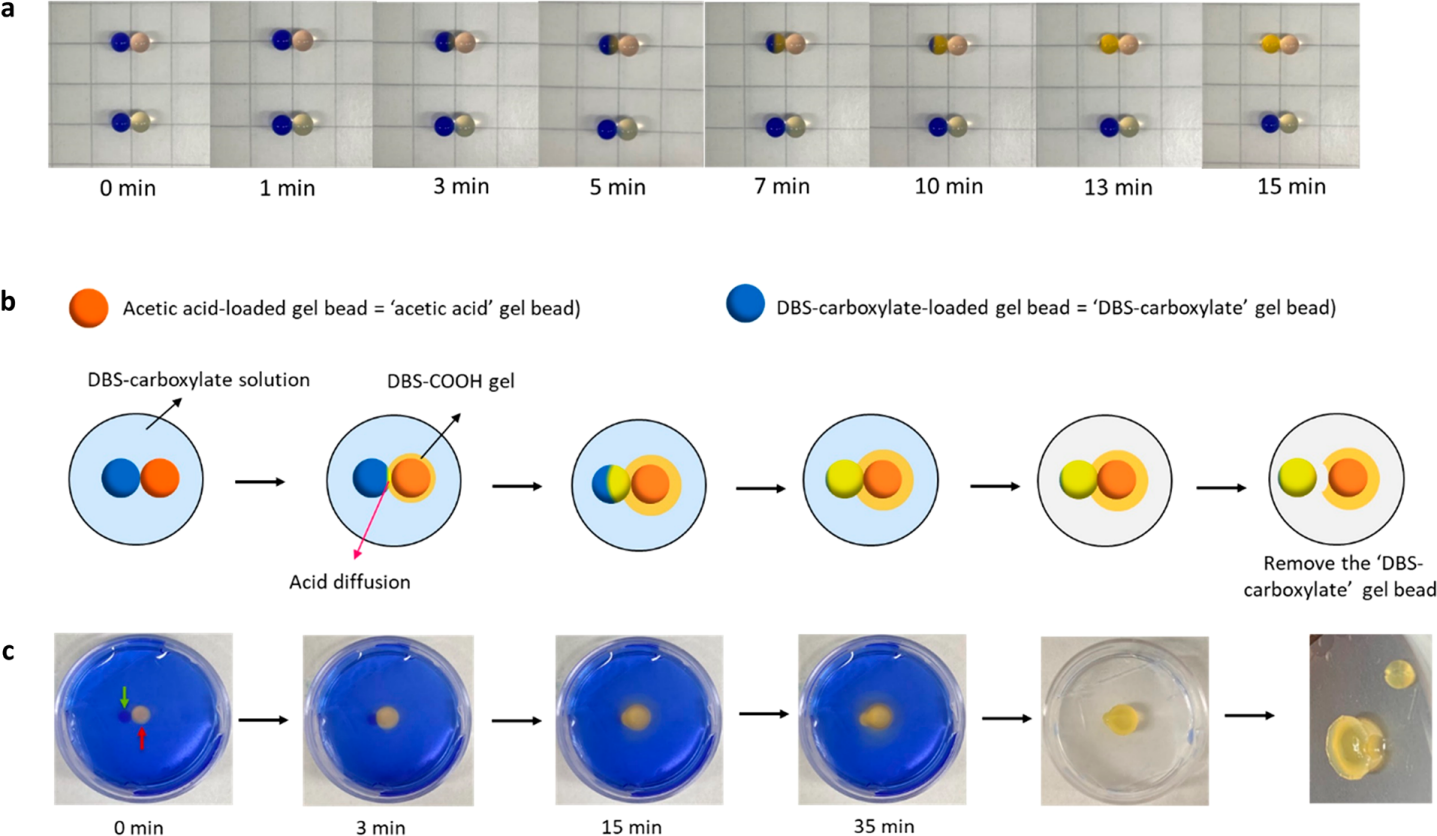
(a) Top: photographs of a 0.062 mmol CH_3_COOH-loaded gel bead and a DBS-carboxylate-loaded gel bead in contact with one another, and bottom: control experiment using a gel bead with no CH_3_COOH. (b) Schematic diagram of the imprinting experiment. Two gel beads were immersed in DBS-carboxylate (0.3 wt %/vol, 3 mL) in a 3.5 cm Petri dish. The blue bead was loaded with DBS-carboxylate (0.3 wt %/vol) and the orange-yellow bead with CH_3_COOH (0.062 mmol). Acid diffusion causes the growth of a DBS-COOH shell but not at the interface between beads. At the end of the experiment, the DBS-carboxylate bead can be removed to leave an imprint. (c) Photographs of the experiment described in the schematic diagram in part (b).

We next reasoned that these gel beads might be able to interact with one another in other, more sophisticated ways (Figure 5).^14^ To probe this possibility, DBS-CONHNH_2_/agarose gel beads were loaded with CH_3_COOH (1 M) in the presence of thymol blue by soaking, while others were loaded with basified DBS-carboxylate solution (0.3 wt %/vol, pH = 11.7). The acid-loaded bead was pink, while the DBS-carboxylate loaded bead was blue. The two beads were placed in gentle contact with one another on top of parafilm in the absence of solvent, and the acid diffused directly through the attachment point between them, from the acid-loaded gel bead into the DBS-carboxylate-loaded gel bead (Figure 5a, top). The pH change of this latter bead was visualized by its blue-yellow color change over ca. 15 min.

We then investigated whether this could help shape and pattern hydrogel objects. A “DBS-carboxylate” gel bead was placed in contact with the acid-loaded gel bead in a basified solution of DBS-carboxylate (0.3 wt %/vol) (Figure 5b and c). After a few minutes, the DBS-carboxylate-loaded gel bead underwent a pH change at the contact region, as visualized by the indicator as CH_3_COOH diffused into it. At the same time, the acid also diffused outward into the surrounding DBS-carboxylate solution with a DBS-COOH gel forming around the acid-loaded gel bead in the usual way. However, there was no DBS-COOH gel assembly around the DBS-carboxylate loaded gel bead, as this was not releasing acid; indeed, it was acting, as a sink, to absorb it, presumably via protonation of the DBS-carboxylate in the bead. Furthermore, this bead prevented formation and adhesion of the DBS-COOH shell at the point it was in contact with the acid-loaded gel bead. As such, the DBS-carboxylate loaded bead could be manually removed leaving an “imprint” in the acid-loaded bead (Figure 5b and 5c, right).

**Figure 6:**
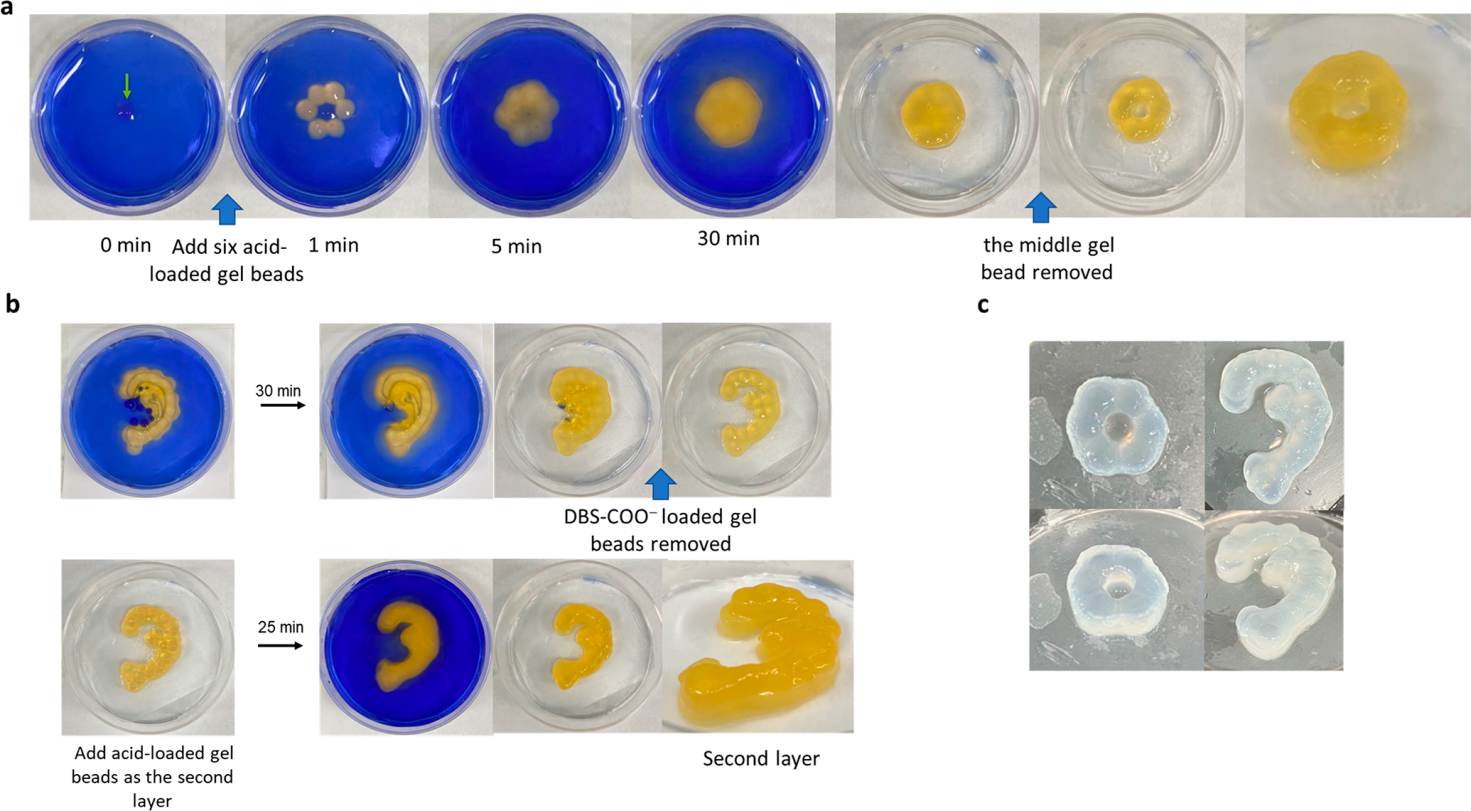
(a) The formation of a ring-donut-like shape from the gel beads loaded with acetic acid (0.062 mmol) or DBS-carboxylate (0.3 wt %/vol) in the 3.5 cm Petri dish containing the DBS-carboxylate (0.3 wt %/vol, 3 mL). (b) The formation of an ear-like shape in a 6 cm Petri dish using layer-by-layer assembly with template beads. (c) Photographs of ring donut and ear shapes after incubating in water for 2 days to wash out indicators.

We reasoned that if DBS-carboxylate-loaded gel beads were embedded within a more complex patterned array of acid-loaded beads, they could, at the end of the experiment, easily be detached, because they do not form their own surface shell of DBS-COOH. They can thus be considered as nonadhesive templating “space-fillers”. Their removal leaves a molded “imprint”, without causing any damage to the remainder of the surrounding DBS-COOH gel. In this way, we could use the diffusion-adhesion approach to create some complex objects (Figure 6).

For example, a DBS-carboxylate-loaded gel bead was placed in the DBS-carboxylate solution (0.3 wt %/vol) in a Petri dish and six acid-loaded gel beads were placed around it (Figure 6a, Figure S33). The acid diffused in all directions including to the bead loaded with DBS-carboxylate, as indicated by the color change. However, DBS-COOH assembly only occurred around the acid-loaded beads, not the central DBS-carboxylate bead, which acts as an acid “sink” as described above. Removing the DBS-carboxylate solution gave a circular gel object. Subsequently, the central “template” gel bead was removed with tweezers, giving rise to a “ring donut” shape, where the central bead has provided an “imprint”.

Even more complex structures such as ear-like shapes (Figure 6b, Figure S34) were fabricated by growth from acid-loaded gel beads, using multiple gel beads loaded with DBS-carboxylate (0.3 wt %/vol) as removable templates to help control and direct the growth of the DBS-COOH shell, giving the final object greater spatial definition. This ear-shaped example also used a second layer of acid-loaded gel beads, via the principles outlined in Figure 4, in order to achieve enhanced three-dimensionality.

### 2.5 Metal Nanoparticle Loading into the Gel Bead Core and Diffusion-Adhesion of NP-Loaded Gels

A unique property of the DBS-CONHNH_2_ gelator is its ability to reduce precious metal salts *in situ* giving rise to metal nanoparticles (NPs) embedded in the gel.^29^ During NP formation, DBS-CONHNH_2_ is converted to DBS-COOH, which still forms a gel; hence, the material retains its integrity.^30^ The resulting NPs are unable to diffuse out of the gel and remain entrapped. It is believed this is a result of the affinity of the naked nanoparticles generated in this way for the carboxylic acid functionalized gel fibers and/or their poor solubility in water. We have previously used this approach to create functional gels with embedded catalytic (PdNPs),^30,31^ antibacterial (AgNPs),^32^ and conductive (AuNPs) NPs.^29^

We exposed the DBS-CONHNH_2_/agarose gel bead to an aqueous solution of AuCl_3_ or AgNO_3_ prior to acid-loading (see Supporting Information). The gel beads turned dark-purple (Au) or orange/yellow (Ag). TEM indicated the presence of NPs with diameters of 5–30 nm (Figure S35). The amount of gold incorporated into the gel beads was quantified by UV–vis spectroscopy at *ca*. 14 μmol of Au/mL gel (Table S5). Given that there is 6.3 μmol of DBS-CONHNH_2_/mL of gel, this demonstrates that each of the two acylhydrazides in the LMWG reduces one metal ion. Acetic acid was then loaded into these gel beads by soaking as previously described. ^1^H NMR (Table S6) indicated that the amount of acid per gel bead was ca. 10% less in the presence of AuNPs than in their absence. This may result from the fact that after NP loading, DBS-CONHNH_2_ has been converted to DBS-COOH, which is less amenable to acid loading. Alternatively, this could result from denser packing of the gel with NPs.

**Figure 7:**
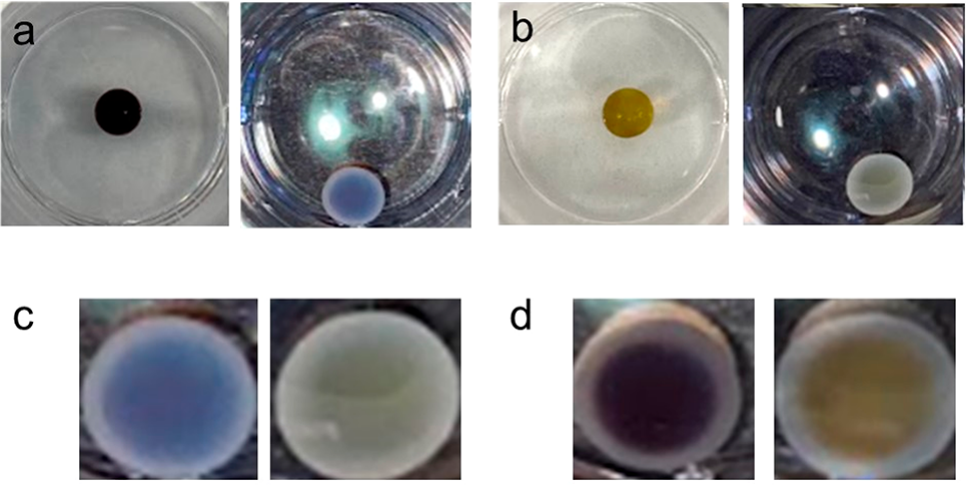
(a) Photographs of DBS-CONHNH_2_/agarose gel bead loaded with AuNPs (left) with a shell of DBS-COOH after 10 min growth (right) in a 3.5 cm Petri dish. (b) Photographs of DBS-CONHNH_2_/agarose gel bead loaded with AgNPs (left) with a shell of DBS-COOH after 10 min growth (right) in a 3.5 cm Petri dish. (c) Core–shell gel beads with a DBS-COOH shell and core loaded with AuNPs (left) or AgNPs (right). (d) Core–shell gel beads with a DBS-COOH/GG shell and core loaded with AuNPs (left) or AgNPs (right). Photos of core–shell gel beads taken with flash to help visualize the structure.

The acid-loaded, AuNP-loaded gel beads were then placed in a gently disturbed DBS-carboxylate solution (0.3 wt %/vol) to produce core–shell gel beads with DBS-COOH as shell. This was repeated in the presence of gellan gum (0.4 wt %/vol). As expected, higher acid loading in the gel bead core produced larger core–shell gel beads with more DBS-COOH assembly (Figures S36–S37). Core–shell gel beads were thus created in which metal NPs are present only in the core (Figure 7). Simple visual observation showed a boundary between core and shell structures with both AuNP (Figure 7a–b) and AgNP (Figure 7c–d) loaded beads. The colored NP-loaded core was more distinct within the shell of DBS-COOH/GG (Figure 7b and d) than in the DBS-COOH shell (Figures 7a and c), owing to the greater optical transparency of the gel in the presence of GG. Optical microscopy confirmed the boundary between core and shell (Figure S47). In these materials, each LMWG plays its own unique role in this process—DBS-CONHNH_2_ generates NPs with spatial resolution in the core, while the pH-responsive nature of DBS-COOH allows shell growth with spatiotemporal control.

Thermal stability was assessed in sample vials using tube inversion, with NPs having no impact on the thermal stability. Rheology (Figures S40–S46, Table S8) indicated that the AuNP-loaded DBS-CONHNH_2_/agarose gel was slightly stiffer than without NPs (*ca*. 16600 Pa vs *ca*. 13500 Pa). The two-layer systems with DBS-COOH and DBS-COOH/GG upper layers were slightly softer (*G*′ = *ca*. 10200 and 13140 Pa respectively) but once again were stiffer than the two-layer systems without NPs.

TEM indicated a nanofibrillar network formation. The fibers of the DBS-COOH gel shell were slightly wider (Figure S48), while the DBS-COOH/GG gel shell had long, narrower fibers (Figure S49). TEM images also confirmed that there were no NPs present in the shells, indicating that the NPs were retained inside the gel bead core during shell growth. SEM images of core–shell gel beads showed that the surface of the shell was different from that of the AuNP-loaded core, confirming the existence of a core–shell structure (Figures S50–S55). The surface of the DBS-COOH shell is densely packed and smooth, while a rough surface was observed for the DBS-COOH/GG shell.

**Figure 8:**
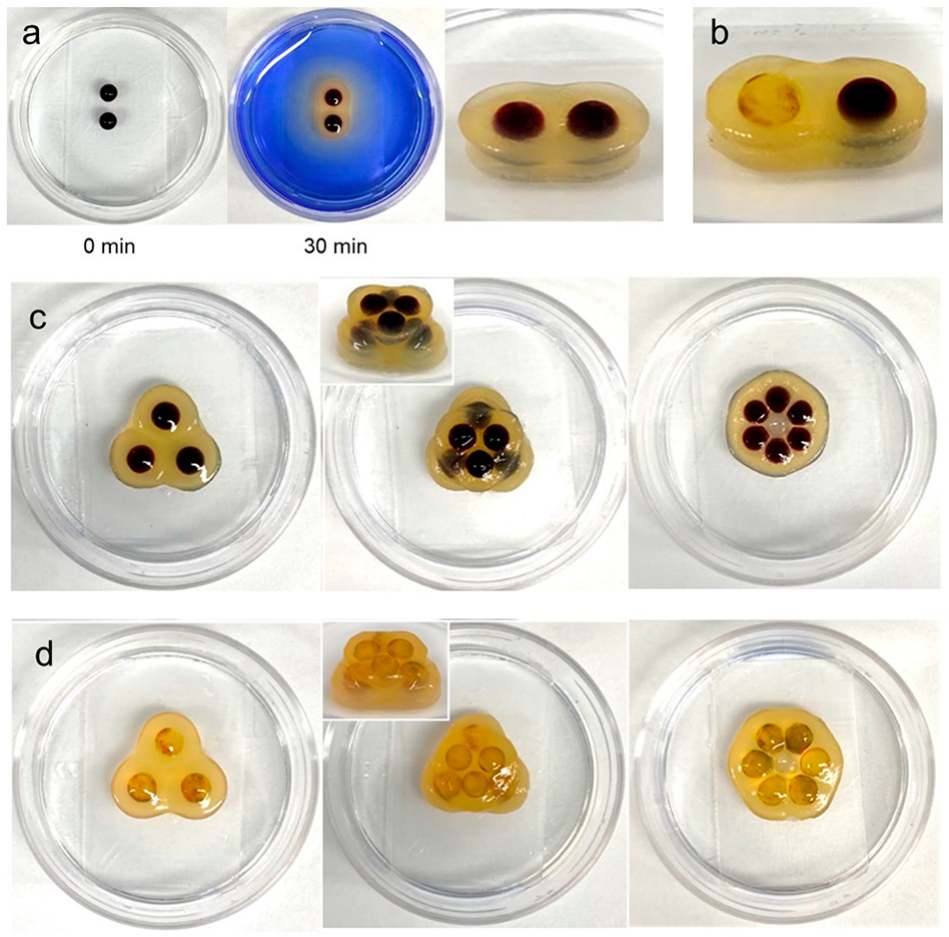
Formation of free-standing hydrogel objects in which the CH_3_COOH-loaded (0.055 mmol/bead) gel beads were preloaded with metal NPs. (a) When two beads were placed in a 3.5 cm Petri dish containing DBS-carboxylate (0.3 wt %/vol, 3 mL), a shell of DBS-COOH grew to create a new NP-loaded object. (b) Result of adhesion between AgNP-loaded gel bead (left) and AuNP-loaded gel bead (right). (c) Formation of (left to right) triangle, double triangle and donut-shaped hydrogels based on acetic acid-loaded gel beads incorporating dark purple AuNPs or (d) orange/yellow AgNPs in 3.5 cm Petri dishes.

We then performed diffusion-adhesion experiments with DBS-CONHNH_2_/agarose gel beads that had been loaded with NPs prior to acid loading. Shaped and patterned 3D gels were obtained in which defined domains of metal NPs are located within a supporting gel matrix created via the DBS-COOH assembly (Figure 8). These materials harness the advantages of both LMWGs, with DBS-CONHNH_2_ enabling *in situ* NP formation and DBS-COOH enabling pH-responsive dynamic shell assembly. The resulting gels demonstrate the sophistication of objects that can be obtained using this simple approach, and emphasize the distinct boundaries between domains, that can be achieved. This type of multidomain gel-in-gel patterning would be challenging to achieve using other methods, such as 3D-printing.

Figure 8 illustrates the assembly of two AuNP-loaded gel beads within a DBS-COOH shell (Figure 8a, Figure S59), and one AuNP bead and one AgNP bead (Figure 8b, Figure S63). Again, the amount of acid loaded into the bead correlated with the growth of the DBS-COOH shell (Figures S57 and S58). Figure 8c illustrates the assembly of triangle (left), double triangle (center), and donut-shaped (right) objects containing AuNPs (top), and Figure 8d incorporates AgNPs into equivalent objects (see also Figures S60–S62 and S64–S67). These were fabricated applying the methods described previously, only using NP-loaded gel beads. In principle, a variety of patterns using gel beads containing AuNPs or AgNPs at defined locations can easily be fabricated. Furthermore, NPs based on other precious metals, such as Pd, or even unloaded gel beads, could also be incorporated into these types of objects.

It is known that metal NPs can encourage stem cell growth and differentiation.^33^ Furthermore, NP-loaded gels can also exhibit conductive, catalytic, or antibacterial properties.^29−32^ Although beyond the scope of this fundamental study, we suggest that the ability to spatially program these NPs within a dynamically assembled 3D gel array opens exciting potential applications in technologies including regenerative medicine, reaction engineering, and the creation of soft electronic devices. For example, tissue growth is a dynamic process in which cells respond to their surrounding medium. By developing shaped gels that also have dynamic spatially controlled properties, we hypothesize that it may be possible to couple the dynamics of both materials and biological systems in order to direct and control tissue growth in a dynamic way in real time.

## 3 Conclusions

In summary, acid-diffusion enables the assembly of core–shell gel beads with different compositions in the core (DBS-CONHNH_2_/agarose) and shell (DBS-COOH with or without gellan gum), the growth of which can be spatially and temporally controlled. If multiple fixed gel beads are allowed to grow DBS-COOH shells in close proximity to one another, the beads adhere to one another within a surrounding shell of gel. In this way, DBS-COOH acts like a mesoscale supramolecular adhesive. By controlling the starting geometry, it is possible to define the structures obtained, and via layer-by-layer methods, 3D objects can be fabricated using this diffusion-adhesion approach.

While acid-loaded beads are adhesive when placed in DBS-carboxylate solution, beads loaded with basic DBS-carboxylate do not grow and act as a sink for the diffusing protons. Hence, they do not adhere to the remainder of the growing gel object. In this way, they can act as templates and enable the “imprinted” fabrication of more complex three-dimensional multigel, multidomain architectures.

Loading the DBS-CONHNH_2_/agarose gel beads with metal NPs prior to acid loading suspends the NPs at precisely defined locations within a larger gel and is an effective way of fabricating multidomain, multifunctional gel objects.

These innovative supramolecular gel objects contain two different LMWGs—DBS-CONHNH_2_ that enable the core to be loaded with NPs, while the pH-responsiveness of DBS-COOH allows dynamic growth of the gel shell onto the core.

We reason that this practically simple diffusion-adhesion approach to assemble complex multidomain gels offers potential for the formation of dynamic gel objects that might see application in high-tech settings, including regenerative medicine or soft nanoscale electronics. In particular, we see the potential to couple the dynamic growth of such hydrogels with the dynamic growth of human stem cells. Research into the applications of these materials is ongoing.

## Supporting Information Available

Detailed description of all experimental methods, additional characterization data, and further details of diffusion-adhesion growth experiments. (PDF)
