# Supplementary material for: Fabricating Shaped and Patterned Supramolecular Multigelator Objects via Diffusion-Adhesion Gel Assembly
Source: J Am Chem Soc. 2023 Oct 27;145(44):24061–70. doi: 10.1021/jacs.3c07376 (PMC10636748; doi:10.1021/jacs.3c07376)
Supplement: Supplementary file 1 — ja3c07376_si_001.pdf [file ja3c07376_si_001.pdf]

Fabricating Shaped and Patterned Supramolecular Multi-Gelator Objects via Diffusion-Adhesion Gel Assembly

Chayanan Tangsombun and David K. Smith*

Department of Chemistry, University of York, Heslington, York, YO10 5DD, UK

SUPPLEMENTARY INFORMATION

S1 General experimental method

S2 Fabrication and characterization of core-shell gel beads

S2.1 Gel preparation
S2.2 NMR studies
S2.3 Infrared (IR) spectroscopy
S2.4 Thermal stability studies
S2.5 Rheology
S2.6 Scanning Electron Microscopy

S3 Fabrication of hydrogel objects via diffusion-adhesion

S3.1 Acid diffusion studies from an immobilized gel bead
S3.2 Patterned gel objects
S3.3 Imprinting in diffusion-adhesion gel assembly

S4 Fabrication and characterization of core-shell gel beads incorporating NPs in the gel bead core

S4.1 Uptake of $AuCl_3$
S4.2 Growing symmetric spherical shell on AuNPs-loaded core gel bead
S4.3 NMR assays
S4.4 Infrared (IR) spectroscopy
S4.5 Thermal stability studies
S4.6 Rheology
S4.7 Optical Microscopy
S4.8 Transmission Electron Microscopy
S4.9 Scanning Electron Microscopy
S4.10 Growing a symmetric spherical shell on AgNPs-loaded core gel bead

S5 Fabrication of hydrogel objects via diffusion-adhesion with NP-loaded gel beads

S5.1 Acid diffusion from NP-looaded gel beads
S5.2 Diffusion-adhesion of NP-loaded gel beads

S6 References

S1 General experimental method

Chemicals were purchased from standard chemical suppliers for synthesis and analysis. Gellan gum was purchased from Alfa Aesar. The two low-molecular-weight gelators, DBS-CONHNH$_2$ and DBS-COOH, were prepared through simple two-step syntheses as described in our previous reports.[1,2] ^{1}H-NMR were recorded on a Jeol 400 spectrometer (^{1}H 400 MHz). Samples were prepared as solutions in deuterated NMR solvents (DMSO-d$_6$ or D_2O) and chemical shifts (δ) are quoted in parts per million (ppm). A Bruker 500 (^{1}H 500 MHz) was used for kinetics experiments. Mass spectrometry was performed by the University of York Mass Spectrometry Service. IR spectra were recorded on a PerkinElmer Spectrum Two FT-IR spectrometer. Optical microscopy images were obtained using a Zeiss Stereo Lumar V12 microscope fitted with AxioCam MRc 5 digital camera. TEM images were recorded on a FEI Tecnai 12 G^2 fitted with a CCD camera. SEM images were obtained from a Jeol JSM 6490LV scanning electron microscope. T_{gel} values was obtained using a high-precision thermoregulated oil bath. Rheological measurements were recorded using a Malvern Instruments Kinexus Pro+ rheometer fitted with a 20 mm parallel plate geometry. UV-Vis absorbance was measured on a Shimadzu UV-2401 PC spectrophotometer. Gel bead sizes and nanoparticle diameters were determined using *ImageJ* software.

S2 Fabrication and characterization of core-shell gel beads

S2.1 Preparation of gel

Preparation of DBS-CONHNH$_2$/agarose gel bead core: DBS-CONHNH$_2$ (3 mg, 0.3% wt/vol) was suspended in water (1 mL) and sonicated for 1 min. This suspension was then added to agarose (10 mg, 1% wt/vol) and sonicated for 5 min. The mixture was heated with a heat gun until complete dissolution. The hot solution was added dropwise (20 µL/drop) to ice cold paraffin oil (50 mL) without stirring. The droplets were left undisturbed overnight. The gel beads were then isolated and transferred into petroleum ether (50 mL, 30 min) to remove the residual of paraffin oil, then EtOH (50 mL, 30 min) and water (50 mL, 30 min). The gel beads were then washed with water multiple times. The gel beads were stored in water and their standard size was 3.0-3.5 mm.

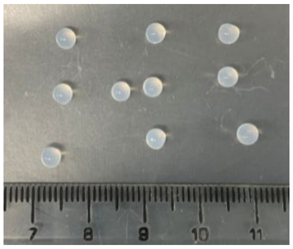

Figure S1 DBS-CONHNH$_2$/agarose gel beads.

Optimisation of Core-Shell Bead Fabrication: We decided to use the DBS-CONHNH$_2$:agarose gel beads as a scaffold for the growth of a DBS-COOH[25] shell. To achieve this, the gel beads were loaded with an acid (fuel) and placed in a vial containing a DBS-carboxylate solution. In an initial unoptimized experiment to test the principle, gel beads were soaked in citric acid (1 M, 3 mL) for 30 min and transferred to an aqueous solution of basified DBS-carboxylate (0.3% wt/vol, 5 mL, pH = 11.2). After a few minutes, the appearance of an opaque spherical shell was observed, with the bead increasing in diameter over time (Fig. 2). The growth of this opaque shell is associated with the self-assembly of the DBS-COOH gelator network onto the surface of the bead – this occurs as acid diffuses from the gel bead and protonates the DBS-COOH, triggering self-assembly at the bead surface.

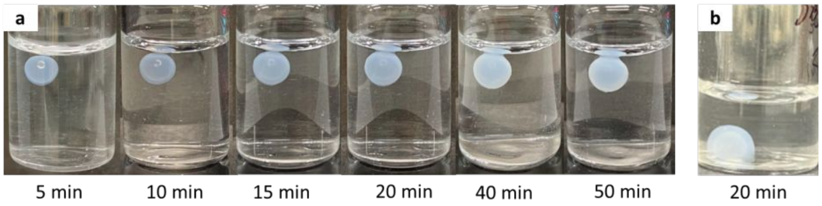

Figure S2 Photographs of shell growth, when a DBS-CONHNH$_2$/agarose gel bead loaded with citric acid (1 M) was placed in DBS-carboxylate solution. (a) Gel bead with air bubble and (b) gel bead without air bubble,

To optimise the objects and simplify the system, we changed from citric acid (pK_{a1} = 3.1) to acetic acid (pK_a = 4.75). However, the major problem was that with either acid, in order to generate a symmetric spherical shell, it was necessary that the gel bead 'floated' within the water in the vial. In our preliminary study, the presence (by chance) of an air bubble in some of the gel beads facilitated this (Fig. S2a). In the absence of an air bubble, the gel beads sank to the bottom of the vial, and the growth of the DBS-COOH shell only occurred on the part of the bead exposed to the solution, leading to an asymmetric shell (Fig. S2b). A number of approaches were therefore used to try and optimise this process:

(i) A test tube with a rounded base was used. It was hoped that even if the gel bead settled, this would help shape the growth, but in reality, wherever the gel bead contacted the glass surface, growth of the DBS-COOH shell was inhibited.
(ii) The solution was slowly stirred with a small stirrer bar. It was hoped this would gently move the bead allowing homogeneous growth, but the loaded gel bead tended to sink, then was destroyed on hitting the stirrer bar.
(iii) The viscosity of the aqueous solution was modified by adding gellan gum (GG). It was hoped to find a viscosity at which the bead would be suspended in the liquid. However, it proved impossible to reproducibly achieve this, with the bead tending to either sink to the bottom or sit at the air-water interface. In both cases, a roughly hemispherical shell resulted.
(iv) The sample vial was gently agitated using occasional gentle disturbance by hand – this led to reproducible spherical gel beads with a good degree of DBS-COOH shell growth.
(v) Further optimisation involved the use of a mechanical shaker, such that the beads remained in constant slow motion (see next section).

Growing a symmetric spherical shell using mechanical shaker: The DBS-CONHNH$_2$/agarose gel beads were prepared as described in the section above. To load the gel beads with acid, ten gel beads were isolated and added into each vial containing the different concentrations of acetic acid solution (3 mL, 1M, 0.5 M, 0.25 M or 0.1 M) for 30 minutes. To prepare the solution of DBS-carboxylate (0.3% wt/vol), DBS-COOH (15 mg) in water (5 mL total) was dissolved with NaOH (240 μL, 0.5 M) then sonicated until complete dissolution. An aliquot of 2 mL of DBS-carboxylate solution (0.3% wt/vol) was added to each well of a 24 well-plate and then placed on a shaker (Heidolph™ Shaker Vibramax 110), with a speed of 325 rpm. An acid-loaded gel bead was gently moved onto a filter paper to remove acid from the surface and then transferred into each well. After each time point, the DBS-carboxylate solution was removed, and the core-shell bead was washed with water. Finally, the core-shell bead was stored in water. For the solution of DBS-carboxylate (0.3 % wt/vol) with gellan gum (0.4% wt/vol), DBS-COOH (15 mg) in water (3 mL total) was dissolved with NaOH (240 μL, 0.5 M) then sonicated until complete dissolution, followed by the addition of gellan gum (2 mL, 1% wt/vol). This solution (2 mL) was added into each well and disturbed with a speed of 485 rpm. For 0.25 M and 0.1 M acid, we could not obtain core-shell gel beads - likely due to the greater movement of the beads making it more difficult for these lower concentrations to establish the required pH gradient.

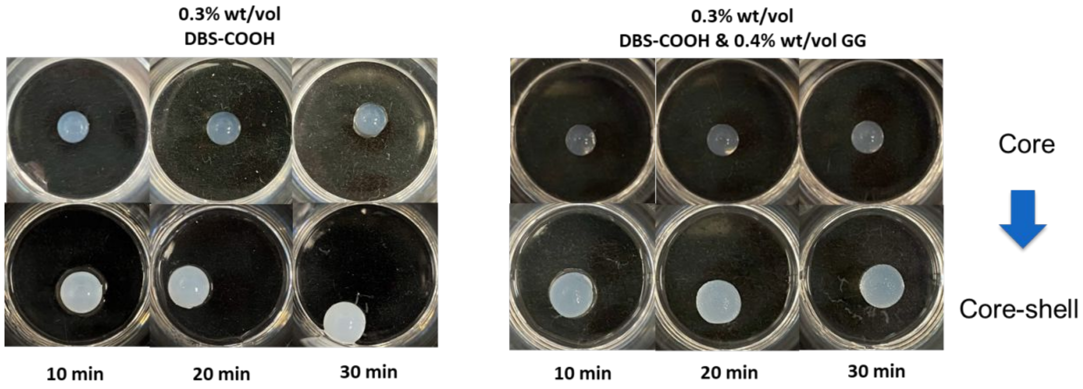

Figure S3 Photographs of core-shell gel beads (below), compared with DBS-CONHNH$_2$/agarose gel bead core (above). The cores were prepared by immersing gel beads in acetic acid (1 M, 3mL, 30 min)

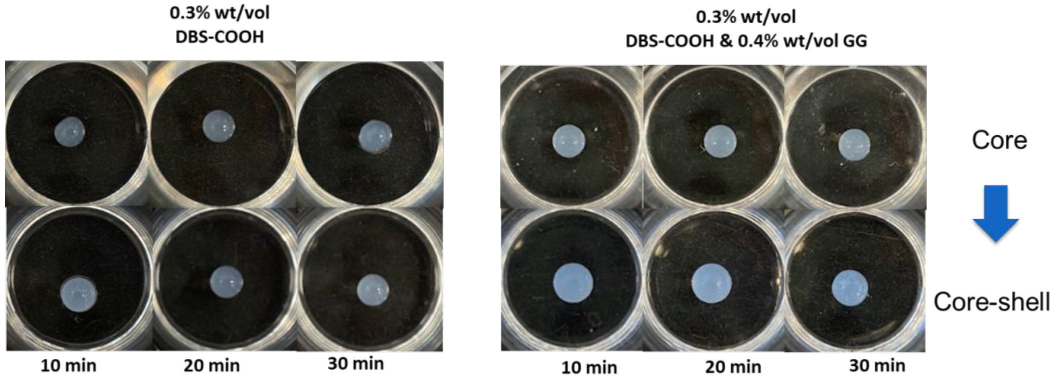

Figure S4 Photographs of core-shell gel bead (below), compared with DBS-CONHNH$_2$/agarose gel bead core (above). The cores were prepared by immersing gel beads in acetic acid (0.5 M, 3mL, 30 min)

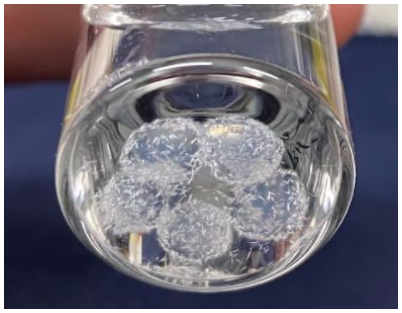

Figure S5 DBS-CONHNH_2/agarose/DBS-COOH/ GG core-shell gel bead stored in water for four days, showing partial crystallisation of the shell.

Preparation of DBS-COOH, gellan gum or DBS-COOH/gellan gum gels: DBS-COOH (3 mg, 0.3% wt/vol) was suspended in water (0.84 mL) and NaOH (60 µL, 0.5 M). This was sonicated to complete dissolution, and then added into another sample vial containing acetic acid (10 M, 0.1 mL) as quickly as possible to aid mixing. For gellan gum (0.4% wt/vol), water (0.9 mL) was added to gellan gum (4 mg) and this was stirred to completely dissolve. The solution was transferred to a new sample vial containing acetic acid (10 M, 0.1 mL) as quickly as possible to aid mixing. For DBS-COOH/gellan gum (0.3% wt/vol of LMWG and 0.4% wt/vol of PG), 3 mg of DBS-COOH was dissolved in water (0.84 mL) and NaOH (60 uL, 0.5M). The DBS-carboxylate solution was transferred and mix with gellan gum (4 mg). This mixture was stirred to complete dissolution, before it was mixed with acetic acid (10 M, 0.1 mL) to form gels. These samples were then left to allow gel formation.

Preparation of DBS-CONHNH_2 gels: DBS-CONHNH_2 (3 mg, 0.3% w/v) was suspended in water (1 mL). The mixture was sonicated and then heated with a heat gun until complete dissolution. This was left undisturbed overnight to allow gel formation.

Preparation of agarose gels: Agarose (13 mg, 1.3% wt/vol) was suspended in water (1 mL) and sonicated for 5 min. The suspension was heated with a heat gun until complete dissolution and left undisturbed overnight.

Preparation of DBS-CONHNH_2/agarose: DBS-CONHNH_2 (3 mg, 0.3% wt/vol) was suspended in water (1 mL) and sonicated for 1 min. This suspension was then added to agarose (10 mg, 1% wt/vol) and sonicated for 5 min. The mixture was heated with a heat gun until complete dissolution. This was left undisturbed overnight to allow gel formation, giving 1 mL gel. To prepare 0.5 mL of DBS-CONHNH_2/agarose gel for core-shell gel, the mixture of agarose (1% wt/vol) and DBS-CONHNH_2 (0.3% wt/vol) in water (1 mL) was heated with heat gun until complete dissolution. The hot solution (0.5 mL) was transferred to a new vial and left to stand to form a gel.

S2.2 ^{1}H-NMR studies

Quantification of DBS-CONHNH$_2$ incorporated in a DBS-CONHNH$_2$/agarose gel bead: The DBS-CONHNH$_2$/agarose gel beads were prepared in the same way as described in Section 2.1. Ten DBS-CONHNH$_2$/agarose gel beads were isolated and dried in a vacuum oven. DMSO-d6 (0.7 mL) was added to dissolve the dried gel beads and sonicated for 30 minutes. Acetonitrile (2 µL) was added into the sample solution as internal standard. The ^{1}H NMR spectrum (e.g. Fig. S6) was recorded and the concentration of DBS-CONHNH$_2$ calculated by the comparison of the integrals of DBS-CONHNH$_2$ aromatic peaks (δ = 7.53 and δ = 7.83 ppm) with that of acetonitrile peak (δ = 2.07 ppm). From the calculation, the amount of DBS-CONHNH$_2$ in the gel bead was 86% (2.57 mg/mL).

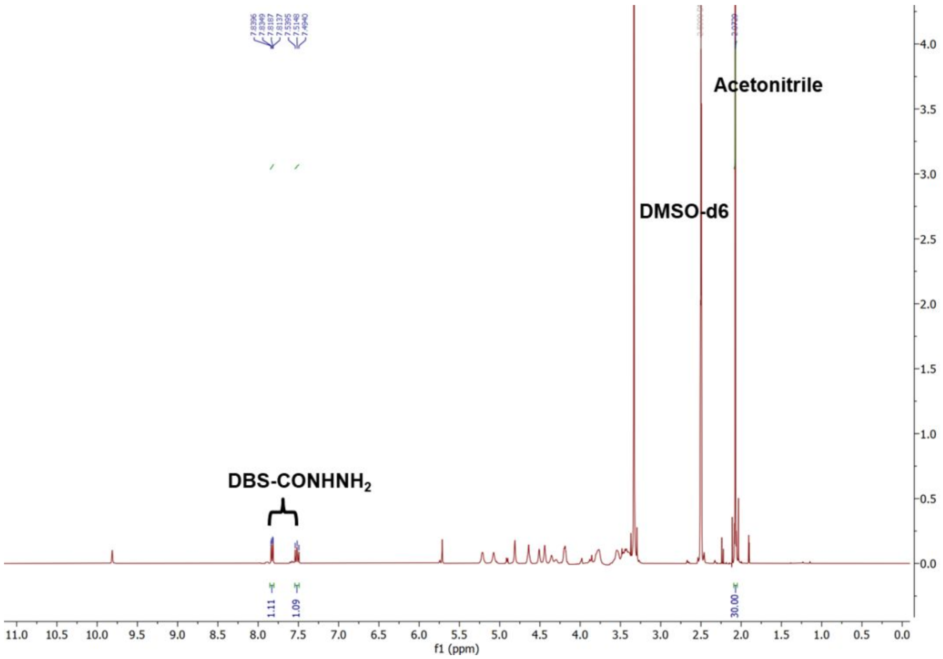

Figure S6 ^{1}H-NMR spectrum of ten dried DBS-CONHNH$_2$/agarose gel beads. Solution was spiked with acetonitrile to quantify DBS-CONHNH$_2$.

Quantification of acetic acid loaded in a DBS-CONHNH$_2$/agarose gel bead: DBS-CONHNH$_2$/agarose gel beads were isolated and immersed in D_2O overnight. After that, ten gel beads were transferred to each vial containing the different concentrations of acetic acid solution prepared from D_2O (1M, 0.5 M, 0.25 M or 0.1 M, 3 mL). After 30 minutes, an aliquot of the acetic acid solution (0.7 mL) was transferred to a vial containing DMSO as internal standard (2 µL). The ^{1}H-NMR spectrum was recorded (Fig. S7), and the concentration of acetic acid calculated left in solution by the comparison of the integrals of acetic acid peak with that of DMSO peak. This experiment was repeated, enabling the average loading of acetic acid per beads to be calculated (Table S1).

Table S1 Summary of acetic acid loaded per gel bead.

initial concentration of acetic acid (M), 3 mL	initial amount of acetic acid (mmol)	integral of acetic acid	integral of DMSO (multiplied by 10)	final amount of acetic acid (mmol)	amount of acetic acid (mmol/ bead)
1.00	3.00	592.83	60.00	2.38	0.062
0.50	1.50	320.59	60.00	1.29	0.021
0.25	0.75	155.09	60.00	0.62	0.013
0.10	0.30	66.12	60.00	0.27	0.003

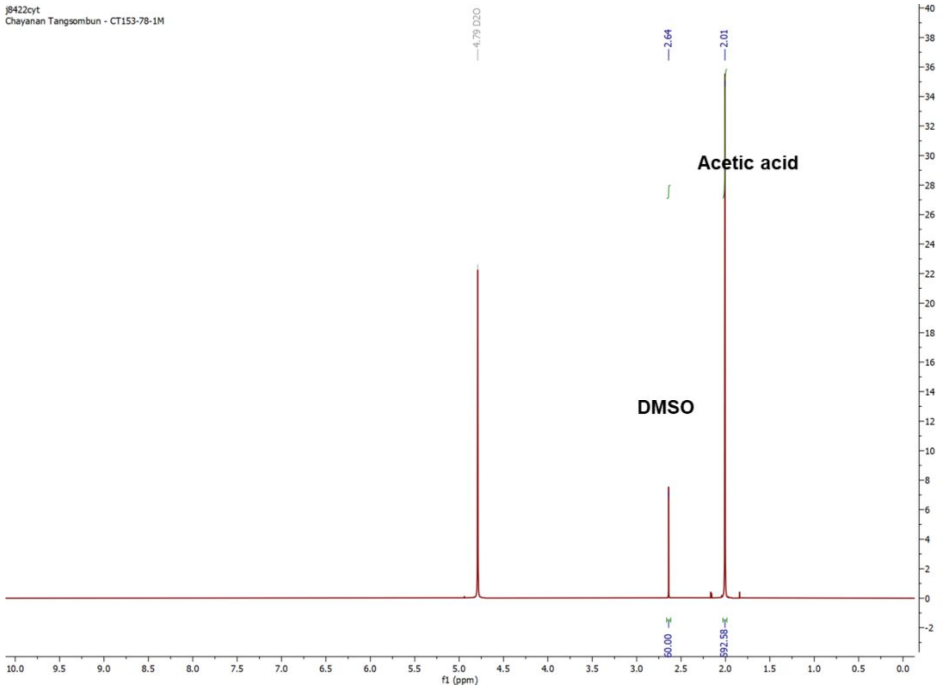

Figure S7 ^{1}H-NMR spectrum of acetic acid aliquots in D_2O, after ten gel beads immersed in 1.0 M of acetic acid (δ = 2.01 ppm) with DMSO as internal standard (δ = 2.64 ppm)

Stability of DBS-CONHNH$_2$ to acetic acid: We had concerns that acid loading may disrupt the DBS-CONHNH$_2$ LMWG network within the bead either as a result of acylhydrazide protonation or acetal hydrolysis. In order to determine the stability of DBS-CONHNH$_2$ during acid loading, ten gel beads loaded with acetic acid from the section above were isolated and transferred into new vials. The beads were placed and dried in air and then in a vacuum oven. DMSO-d6 (0.7 mL) was added to dissolve the dried gel beads and sonicated for 30 minutes. Cyclohexane (3 μL) was added to the sample solution as internal standard. The ^{1}H-NMR spectra of beads loaded with different acetic acid concentrations was recorded, and the amount of DBS-CONHNH$_2$ calculated by the comparison of the integrals of DBS-CONHNH$_2$ aromatic peaks (δ = 7.53 and δ = 7.83 ppm) with that of cyclohexane peak (δ = 1.40 ppm) (see example in Figure S8). The experiment was repeated and the average values reported, demonstrating that DBS-CONHNH$_2$ was retained within the gel beads during acid loading.

Table S2 DBS-$CONHNH_2$ incorporated in the gel beads after loading acid.

Ten gel beads soaked in acetic acid concentration (M)	amount of DBS-$CONHNH_2$ (mg/1 mL)	STD
1.00	2.99	0.24
0.50	2.93	0.20
0.25	3.07	0.19
0.10	3.17	0.21

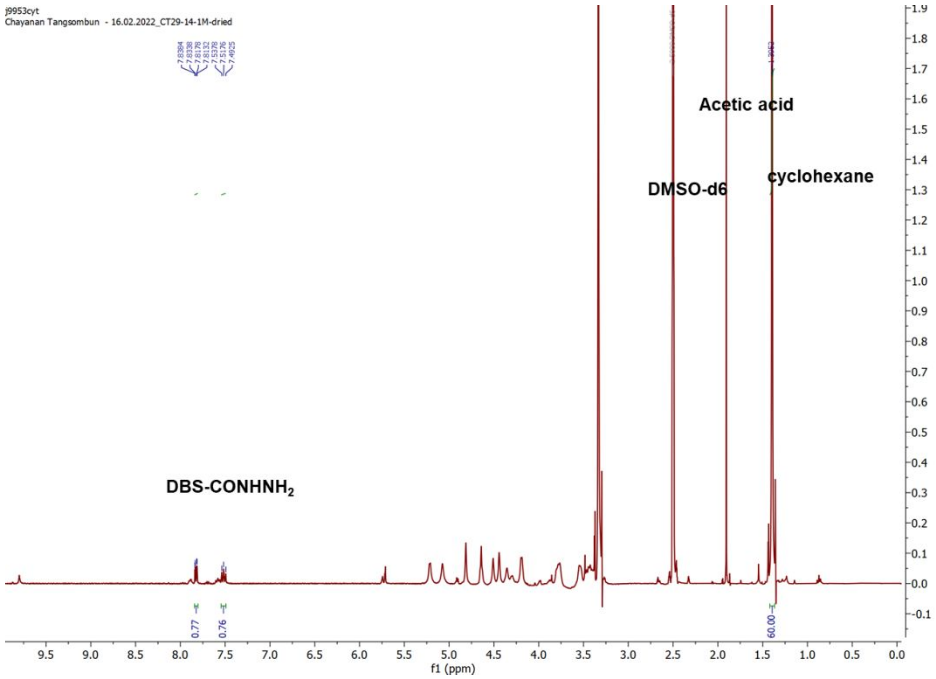

Figure S8 ^{1}H-NMR spectrum of ten dried gel beads immersed acetic acid (1.0 M).

NMR verification of the self-assembly of DBS-COOH shell over time: DBS-$CONHNH_2$/agarose gel beads were isolated and transferred into a vial containing D_2O and this was left overnight. Ten gel beads were then added into acetic acid solution prepared using D_2O (3 mL, 1 M or 0.5 M) for 30 minutes. Five gel beads loaded with acetic acid were transferred into an NMR tube. A suspension of DBS-COOH (0.3% wt/vol) in D_2O (1 mL total) was dissolved by the addition of NaOD (60 μL, 0.5 M) and then sonicated until complete dissolution. The DBS-carboxylate solution (0.6 mL) was transferred into a new vial in the presence of DMSO (2 μL) as internal standard and subsequently added into the NMR tube containing five gel beads loaded with acetic acid. The ^{1}H NMR spectrum was then recorded every 10 minutes for 1 hour and every 30 minutes for 11 hours (Fig. S9). The percentage of mobile components was calculated by comparison of the integrals of DBS-COOH aromatic peaks (δ = 7.66 and 7.92 ppm) with that of the DMSO peak (δ = 2.74 ppm).

To study the effect of gellan gum on DBS-COOH assembly, five gel beads loaded with acetic acid were transferred into an NMR tube. The DBS-carboxylate solution (0.3% wt/vol, 0.6 mL) was transferred into

a new vial containing gellan gum (2.4 mg, 0.4% wt/vol) and DMSO (2 μL) as internal standard. After gellan gum had dissolved, the solution was added into the NMR tube containing five gel beads loaded with acetic acid. The ^{1}H NMR spectrum was then recorded every 10 minutes for 1 hour and every 30 minutes for 11 hours (Fig. S10). The percentage of mobile components was calculated by comparison of the integrals of DBS-COOH aromatic peaks (δ = 7.66 and 7.92 ppm) with that of DMSO peak (δ = 2.74 ppm).

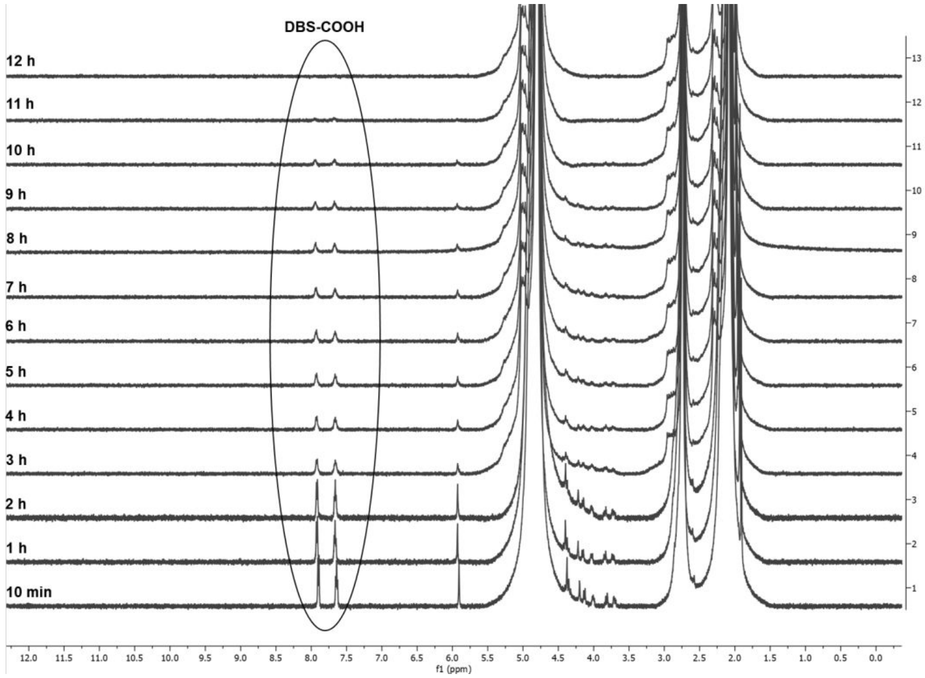

Figure S9 ^{1}H NMR of DBS-COOH after 10 mins and every hour from sample preparation in the presence of five DBS-CONHNH$_2$/agarose gel beads loaded with acetic acid (0.062 mmol/bead) to the solution of DBS-carboxylate.

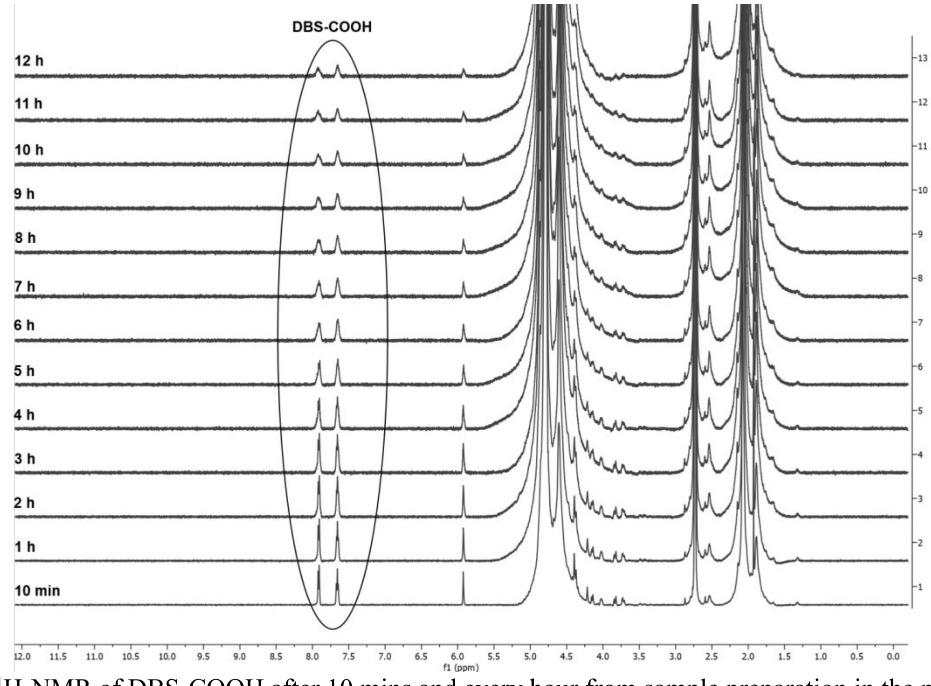

Figure S10 ^{1}H-NMR of DBS-COOH after 10 mins and every hour from sample preparation in the presence of five DBS-CONHNH$_2$/agarose gel beads loaded with acetic acid (0.062 mmol/bead) to the solution of DBS-carboxylate/gellan gum.

Table S3 Percentage of mobile DBS-COOH over time after addition times after addition of five DBS-$CONHNH_2$/agarose gel beads loaded with acetic acid (0.062 or 0.021 mmol/bead) to the solution of DBS-carboxylate (0.3% wt/vol) without or with GG (0.4% wt/vol).

Time (min)	% of mobile DBS-COOH		% of mobile DBS-COOH in the presence of GG	
	0.062 mmol/bead	0.021 mmol/bead	0.062 mmol/bead	0.021 mmol/bead
0	100.00	100.00	100.00	100.00
10	52.99	63.55	70.88	71.42
20	44.96	61.10	67.74	69.83
30	41.11	58.05	65.12	69.40
40	39.63	56.83	64.95	68.79
50	39.19	55.34	63.11	68.09
60	38.50	53.42	61.80	67.39
90	34.83	48.01	56.91	66.87
120	28.37	44.52	53.60	66.17
150	24.35	44.52	49.32	65.99
180	24.09	42.77	45.65	64.95
210	22.35	41.73	42.51	64.77
240	20.78	40.24	39.54	63.99
270	20.43	37.62	36.84	62.59
300	19.03	37.45	34.13	61.98
330	18.59	37.10	31.86	59.88
360	17.37	36.58	29.68	58.31
390	16.32	36.14	27.50	57.00
420	15.71	35.00	25.58	55.34
450	15.10	33.96	24.09	54.47
480	14.67	33.78	22.43	53.51
510	12.57	31.86	20.60	52.81
540	10.82	31.69	19.73	51.33
570	10.04	29.33	18.51	50.54
600	7.68	28.81	17.37	49.84
630	4.80	27.06	16.24	48.62
660	3.67	26.89	15.36	47.66
690	2.71	26.80	14.49	46.88
720	0.00	27.32	14.67	46.53

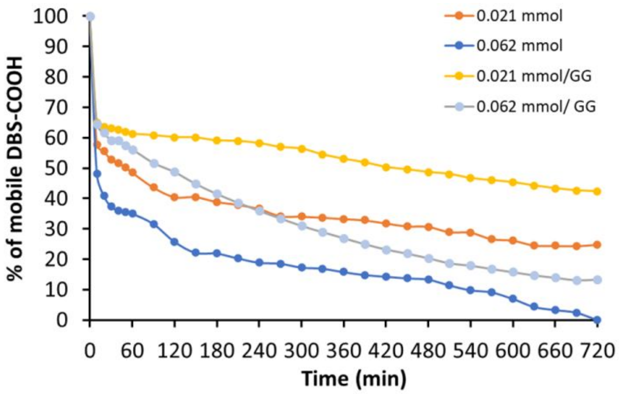

Figure S11 Percentage of mobile DBS-COOH at different times after adding five DBS-$CONHNH_2$/agarose gel beads loaded with acetic acid (0.062 or 0.021 mmol/bead) to a solution of DBS-carboxylate (0.3% wt/vol) without or with GG (0.4% wt/vol).

S2.3 Infrared (IR) spectroscopy

All gels were prepared as described in Section 2.1. The gels were dried under vacuum prior to analysis.

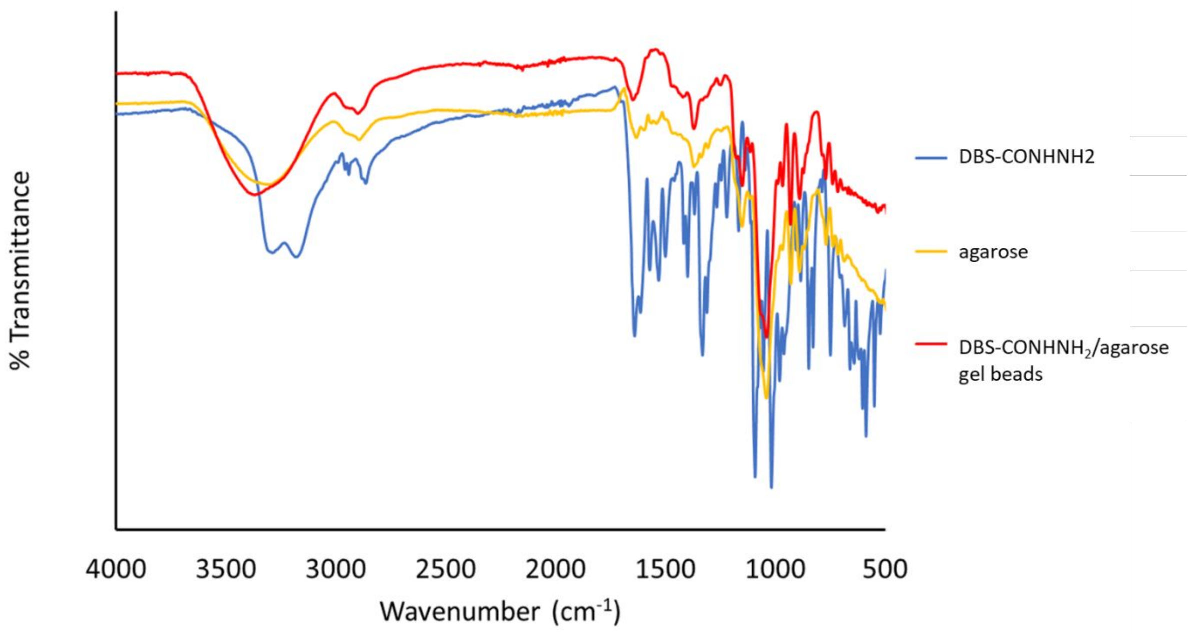

Figure S12 IR spectra of xerogel obtained from DBS-CONHNH$_2$ gel (0.3% wt/vol, blue line), agarose gel (1.3 % wt/vol, yellow line) and DBS-CONHNH$_2$/agarose gel bead (0.3% wt/vol of DBS-CONHNH$_2$ and 1.0% wt/vol of agarose, red line).

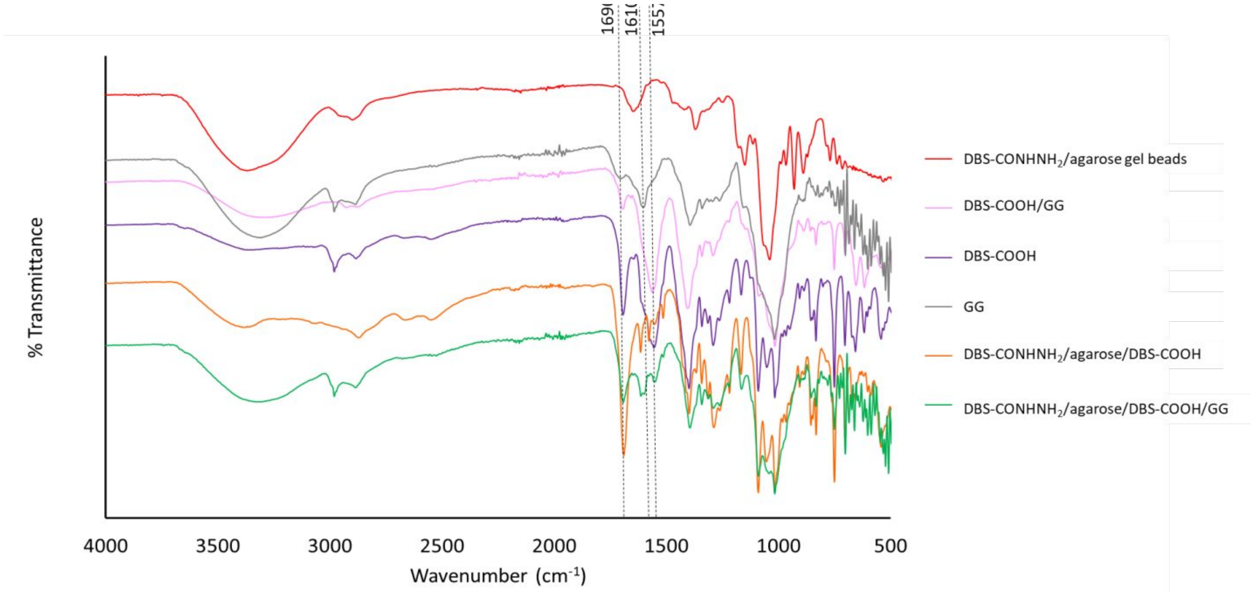

Figure S13 IR spectra of xerogel obtained from DBS-CONHNH$_2$/agarose gel bead (0.3% wt/vol of DBS-CONHNH$_2$ and 1.0% wt/vol of agarose, red line), GG gel (0.4 % wt/vol, grey line), DBS-COOH gel (0.4 % wt/vol, purple line), DBS-COOH/GG gel (0.3% wt/vol of DBS-COOH and 0.4% wt/vol of GG, pink line), DBS-CONHNH$_2$/agarose/DBS-COOH core-shell beads (orange line) and DBS-CONHNH$_2$/agarose/DBS-COOH/GG core-shell beads (green line).

S2.4 Thermal stability studies

All the gels were prepared in 7 ml vials with a circular base (diameter = 2 cm, height = 6 cm) and left overnight, allowing gel formation as described in Section 2.1. For 'core-shell' layered gels, a mixture of agarose (1% wt/vol) and DBS-CONHNH$_2$ (0.3% wt/vol) in water (1 mL) was heated with heat gun until complete dissolution. The hot solution (0.5 mL) was transferred to a new vial, giving 0.5 mL gel in two vials and one of them was used for study. The DBS-CONHNH$_2$/agarose hybrid gel in vial was left to allow gel formation. Acetic acid (0.5 mL, 1 M) was added on top of the gel surface and left for 30 min. The supernatant was then removed, followed by the addition of 0.5 mL DBS-carboxylate solution (0.3% wt/vol) or DBS-carboxylate (0.3% wt/vol) with gellan gum (0.4% wt/vol) solution. The acid diffused from the gel to yield the DBS-COOH gel on top of the DBS-CONHNH$_2$/agarose hybrid gel. All gels were placed in a high precision thermoregulated oil bath with an initial temperature of 25 °C. The temperature was increased by 1 °C/min until 100 °C. The gels were checked by tube inversion method every minute. The temperature (T_{gel}) was recorded when the gel began to run down the side of the vial. These experiments were repeated three times and the average T_{gel} was recorded. Errors are estimated at ±2°C. On heating, the DBS-COOH/GG 'shell' detached itself from the DBS-CONHNH$_2$/agarose more easily than the DBS-COOH 'shell'.

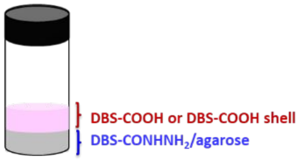

Figure S14 A two-layer gel in a vial

Table S4 T_{gel} values of gels formed by individual gelators, DBS-CONHNH$_2$/agarose gel, DBS-CONHNH$_2$/agarose/DBS-COOH gel, and DBS-CONHNH$_2$/agarose/DBS-COOH/GG gel. The concentration was reported in the unit of % wt/vol.

Gel (1 mL total volume)	DBS-CONHNH$_2$ Loading	Agarose Loading	DBS-COOH Loading	GG Loading	T_{gel}/° C
DBS-CONHNH$_2$	0.3%	-	-	-	88
Agarose	-	1.3%	-	-	99
DBS-CONHNH$_2$/Agarose	0.3%	1.0%	-	-	>100
DBS-COOH	-	-	0.3%	-	77
GG	-	-	-	0.4%	76
DBS-COOH/GG	-	-	0.3%	0.4%	88
DBS-COOH on DBS-CONHNH$_2$/Agarose	0.3%	1.0%	0.3%	-	shell: 77 core:>100
DBS-COOH/GG on DBS-CONHNH$_2$/Agarose	0.3%	1.0%	0.3%	0.4%	shell: 87 core:>100

S2.5 Rheology

Hydrogel samples and 'core-shell' layered hydrogels were prepared in bottomless vials (diameter = 2 cm) as described in Sections 2.1 and 2.4. Measurements were carried out at 25 ℃ using a 20 mm parallel plate and a gap of 2.5 mm. An amplitude sweep was performed in the range of 0.01-100% strain at a frequency of 1 Hz. The frequency sweep was performed between 0.1-100 Hz using a shear strain of 0.15%. For reproducibility, the experiments were repeated three times and average data were used to plot the graph.

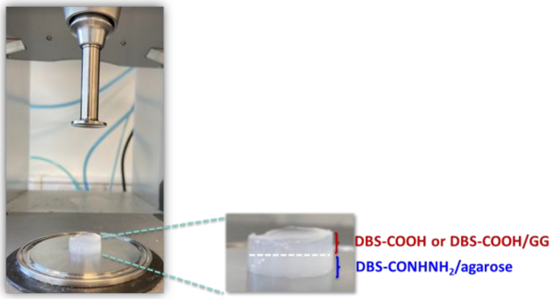

Figure S15 Photograph of core-shell hydrogel prepared for rheology studies.

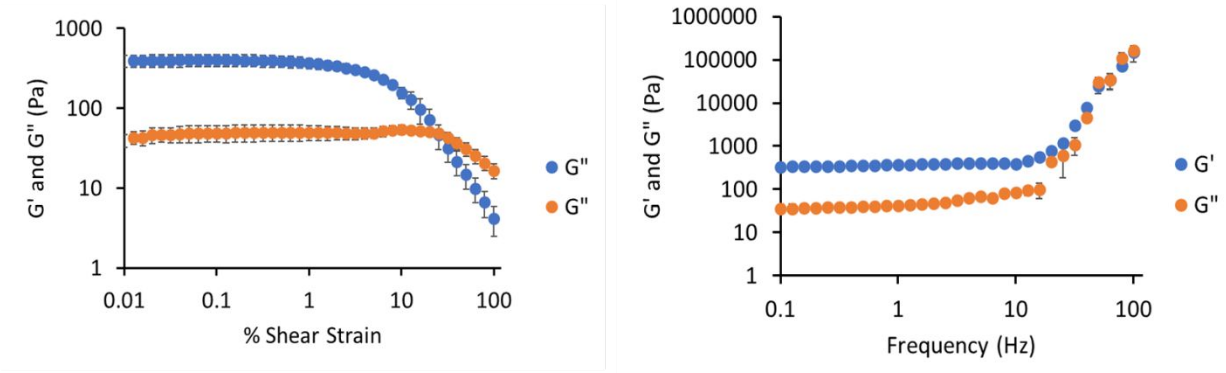

Figure S16 Elastic (G', blue circles) and viscous (G", orange circles) moduli of DBS-COOH hydrogel (0.3% wt/vol) with increasing shear strain (left) and frequency (right).

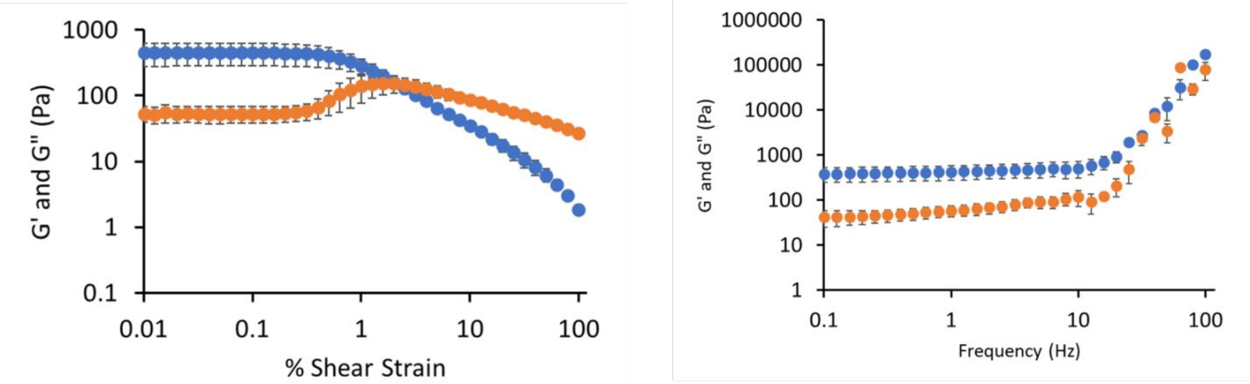

Figure S17 Elastic (G', blue circles) and viscous (G", orange circles) moduli of gellan gum hydrogel (0.4% wt/vol) with increasing shear strain (left) and frequency (right).

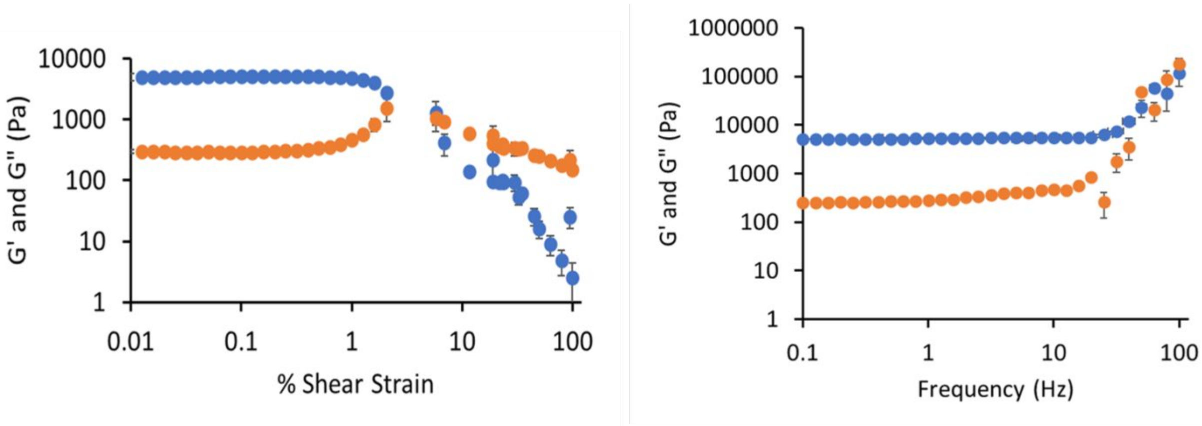

Figure S18 Elastic (G', blue circles) and viscous (G", orange circles) moduli of DBS-COOH/gellan gum hydrogel (0.3% wt/vol DBS-COOH and 0.4% wt/vol gellan gum) with increasing shear strain (left) and frequency (right).

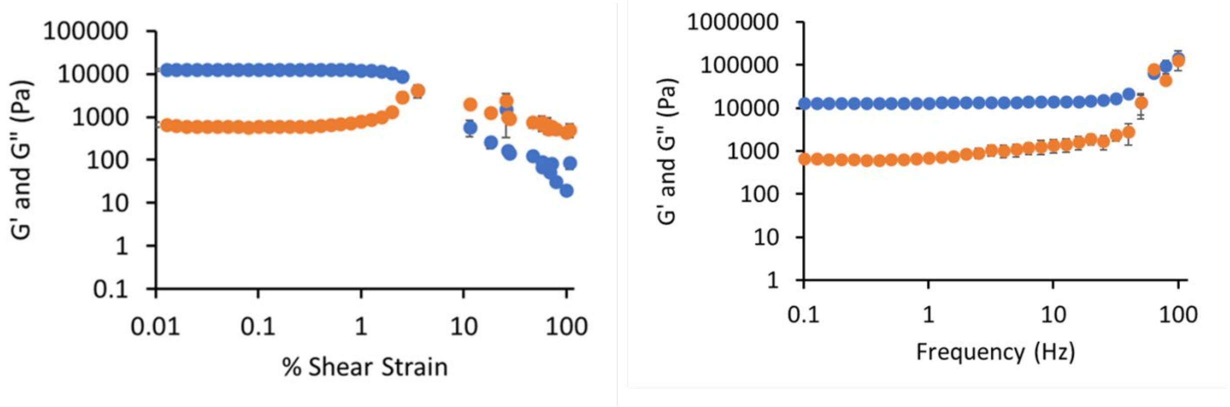

Figure S19 Elastic (G', blue circles) and viscous (G", orange circles) moduli of DBS-CONHNH$_2$/agarose hydrogel (0.3% wt/vol DBS-CONHNH$_2$ and 1.0% wt/vol agarose) with increasing shear strain (left) and frequency (right).

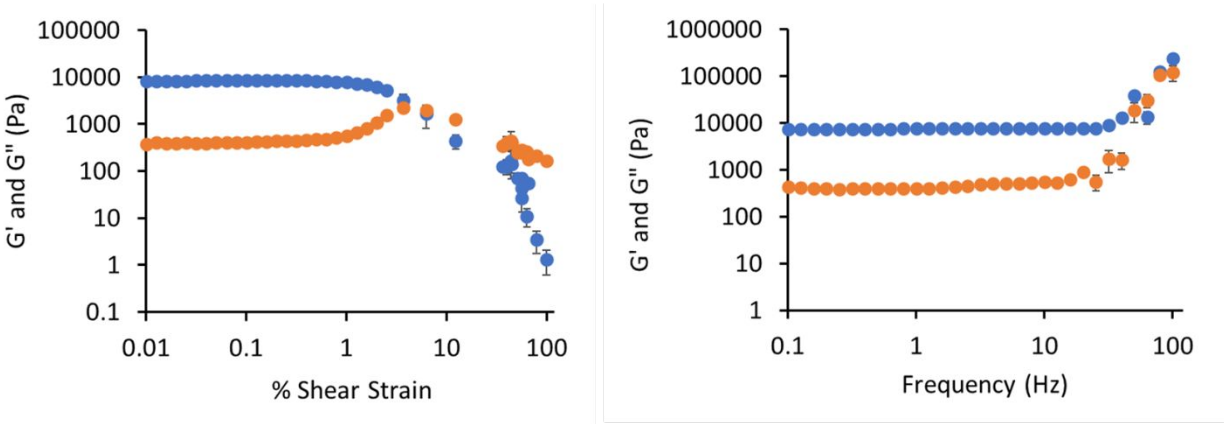

Figure S20 Elastic (G', blue circles) and viscous (G", orange circles) moduli of core-shell layered gel DBS-COOH (0.3% wt/vol) on DBS-CONHNH$_2$/agarose (0.3% wt/vol DBS-CONHNH$_2$, 1.0% wt/vol agarose) with increasing shear strain (left) and frequency (right).

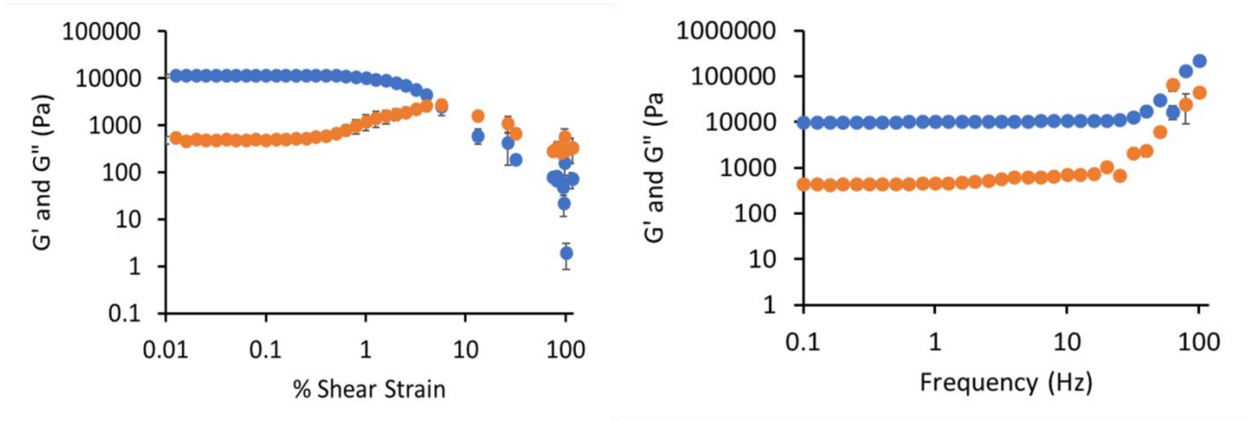

Figure S21 Elastic (G', blue circles) and viscous (G", orange circles) moduli of core-shell layered hydrogel DBS-COOH/gellan gum (0.3% wt/vol DBS-COOH and 0.4% wt/vol gellan gum) on DBS-CONHNH$_2$/agarose hydrogel (0.3% wt/vol DBS-CONHNH$_2$ and 1.0% wt/vol agarose,) with increasing shear strain (left) and frequency (right).

S2.6 Scanning Electron Microscopy (SEM)

The core-shell gel beads were prepared as described in Section 2.1. The samples were dehydrated by washing with a graded series of acetones (50-100%) then critical-point dried in a Polaron E3000 critical-point drier using liquified carbon dioxide. The dried samples were then affixed to SEM stubs and imaged on a Jeol JSM 6490LV scanning electron microscope operating at 5kV.

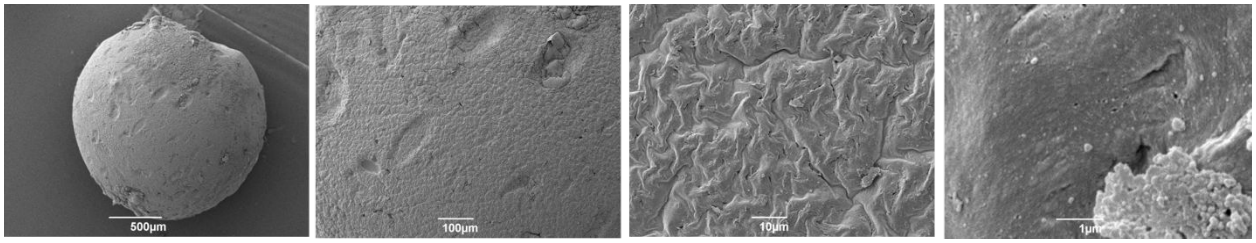

Figure S22 SEM images of DBS-CONHNH$_2$/agarose/DBS-COOH core-shell gel bead and surface. Scale bar from left to right: 500, 100, 10 and 1 μm.

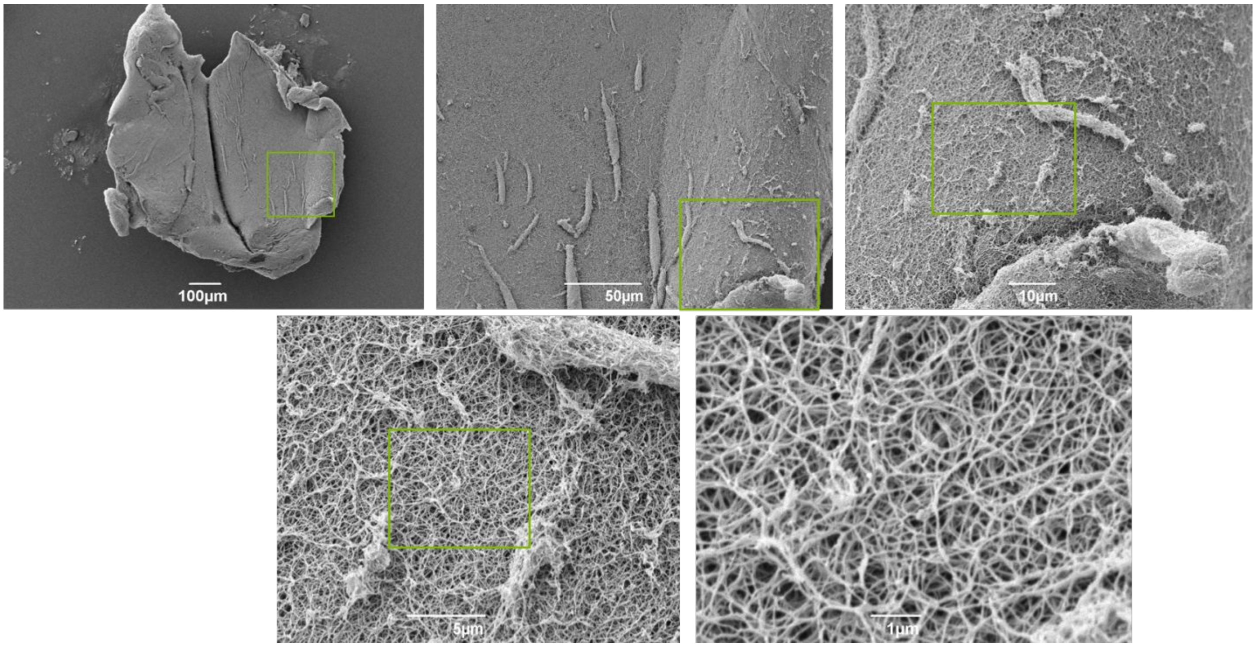

Figure S23 SEM images of DBS-CONHNH$_2$/agarose/DBS-COOH core-shell gel bead cut in a half (zoom in edge). Scale bar from left to right: 100, 50, 10, 5 and 1 μm.

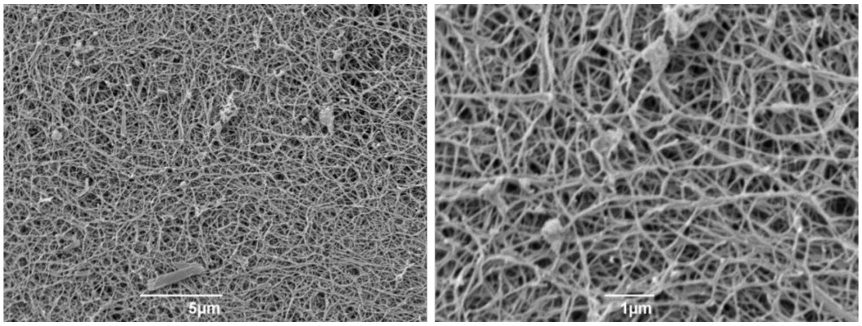

Figure S24 SEM images of DBS-CONHNH$_2$/agarose/DBS-COOH core-shell gel bead cut in a half (zoom in center). Scale bar from left to right: 5 and 1 μm.

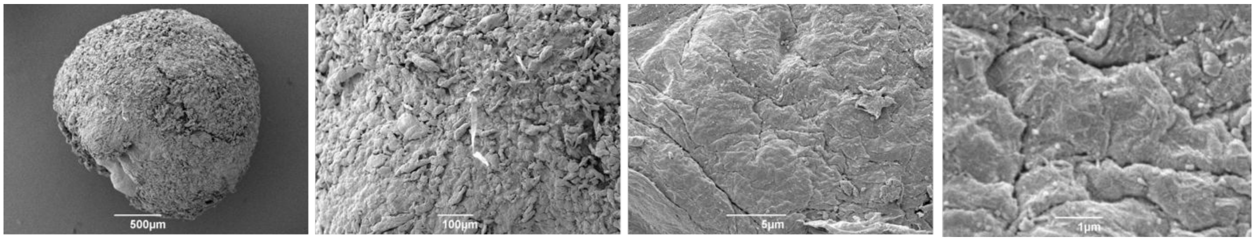

Figure S25 SEM images of whole DBS-CONHNH$_2$/agarose/DBS-COOH/GG core-shell gel bead and surface. Scale bar from left to right: 500, 100, 10 and 1 μm.

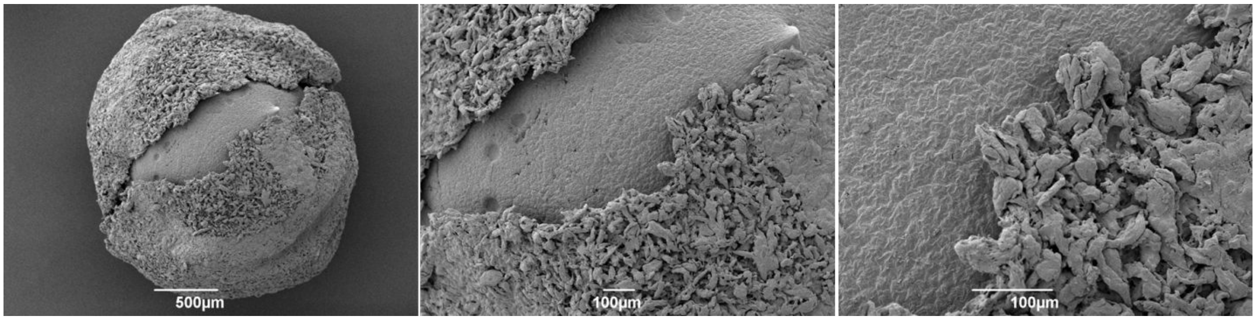

Figure S26 SEM images of broken DBS-CONHNH$_2$/agarose/DBS-COOH/GG core-shell gel bead. Scale bar from left to right: 500 and 100 μm.

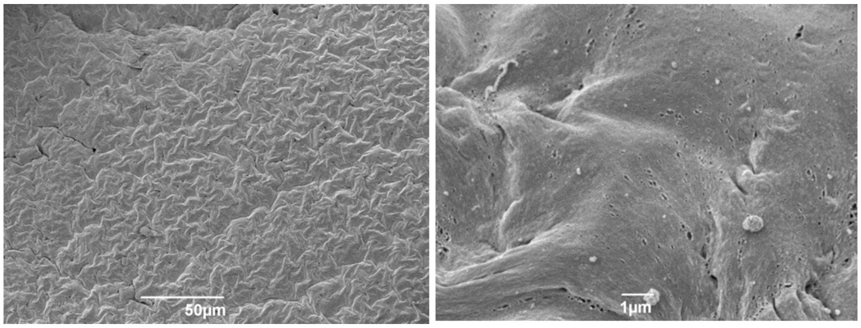

Figure S27 SEM images of core surface from DBS-CONHNH$_2$/agarose/DBS-COOH/GG core-shell gel bead. Scale bar from left to right: 50 and 1 μm.

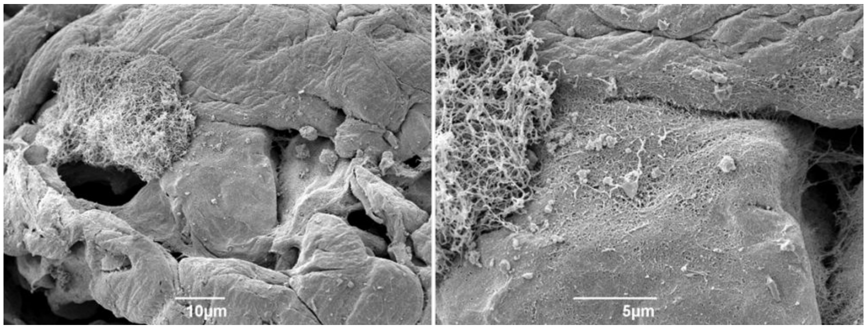

Figure S28 SEM images of broken DBS-CONHNH$_2$/agarose/DBS-COOH/GG core-shell gel bead (zoom in edge). Scale bar from left to right: 10 and 5 μm.

S3 Fabrication of hydrogel objects via diffusion-adhesion

S3.1 Acid diffusion studies from an immobilized gel bead

DBS-COOH (15 mg, 0.3% wt/vol) in water (5 mL total) was dissolved by NaOH (240 μL, 0.5 M) then sonicated until complete dissolution. Thymol blue (15 μL, 1% in EtOH) was added into the solution. The DBS-CONHNH$_2$/agarose gel beads were prepared as described in Section 2.1. Ten gel beads were soaked in vials containing the different concentrations of acetic acid solution (3 mL, 1 M, 0.5 M, 0.25 M or 0.1 M) with thymol blue (15 μL, 1% in EtOH) for 30 minutes. The acid-loaded gel bead was gently moved onto a filter paper to remove acid from the surface. The diameter of the acid-loaded gel bead was recorded and the bead was then transferred to a petri dish that contained the DBS-carboxylate solution (3 mL, 0.3% wt/vol). Photographs were taken at different time points, and the radius of DBS-COOH opaque gel formation was determined using *ImageJ*.

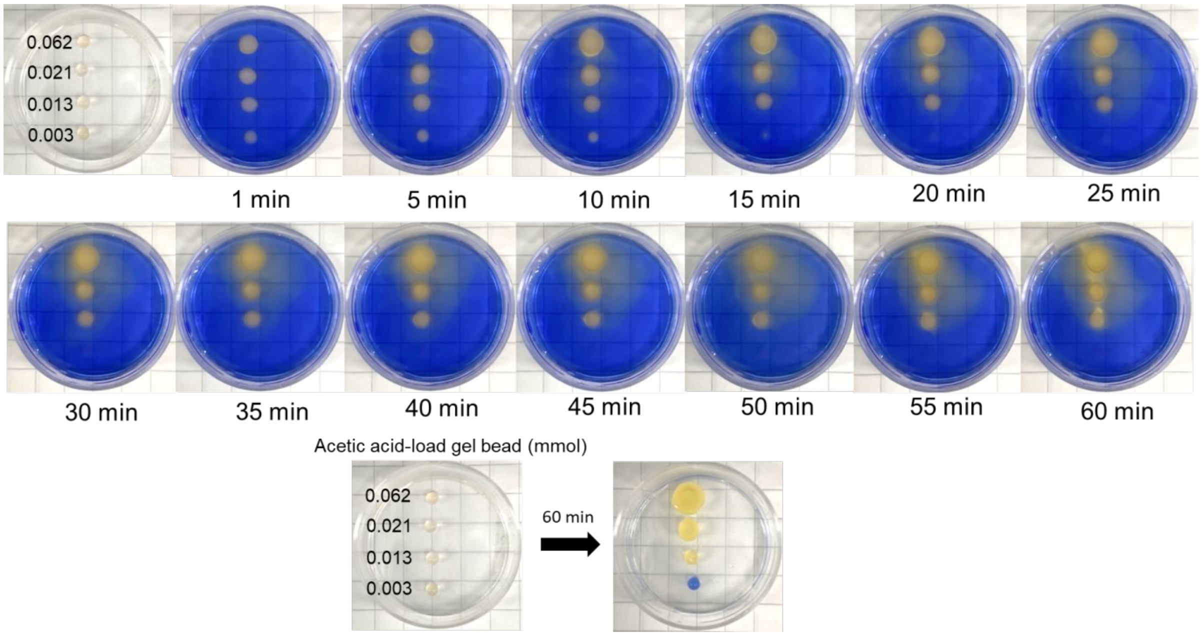

Figure S29 Photographs of growing DBS-COOH gel over time after addition of acetic acid-loaded gel bead into the 3.5 cm petri dish containing the DBS-carboxylate (0.3% wt/vol, 3mL).

S3.2 Patterned gel objects

The solution of DBS-COOH (0.3% wt/vol) was prepared by dissolving DBS-COOH (15 mg) in NaOH (240 μL, 0.5 M) and water (5 mL total), with addition of Thymol blue (15 μL, 1% in EtOH). Ten gel beads were isolated and added into vials containing acetic acid solution (3 mL, 1 M) in the presence of thymol blue for 30 min. Parafilm was placed on the bottom of the petri dish. Acid-loaded gel beads were arranged in patterns on the parafilm and immersed in DBS-carboxylate solution (3 mL, 0.3% wt/vol). For

double triangle fabrication, the triangle hydrogel object was formed in a 3.5 cm petri dish containing DBS-carboxylate solution (0.3% wt/vol, 3 mL) for 30 min. After the spent solution was removed, three more three gel beads were arranged on top of the first triangle layer. The solution of DBS-Carboxylate (0.3%wt/vol, 5 mL) was added into the petri dish. After 25 min, the spent solution was removed, yielding the adhered double triangle object.

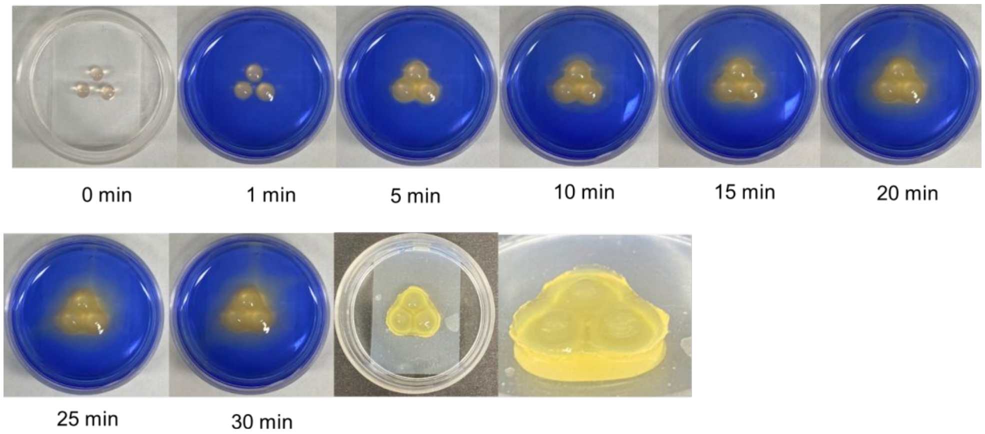

Figure S30 The formation of free-standing hydrogel objects based on three hybrid gel beads loaded with acetic acid (0.062 mmol/bead) in the 3.5 cm petri dish containing the DBS-carboxylate (0.3% wt/vol, 3mL).

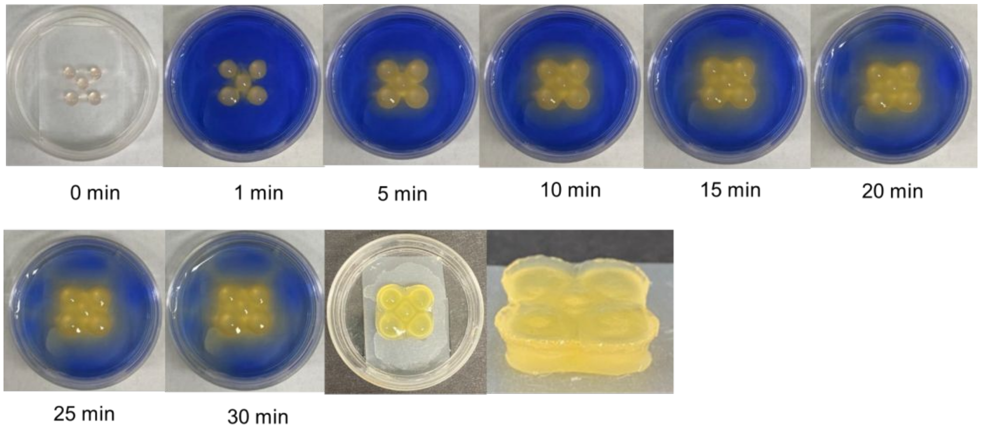

Figure S31 The formation of free-standing hydrogel objects based on five hybrid gel beads loaded with acetic acid (0.062 mmol/bead) in the 3.5 cm petri dish containing the DBS-carboxylate (0.3% wt/vol, 3mL).

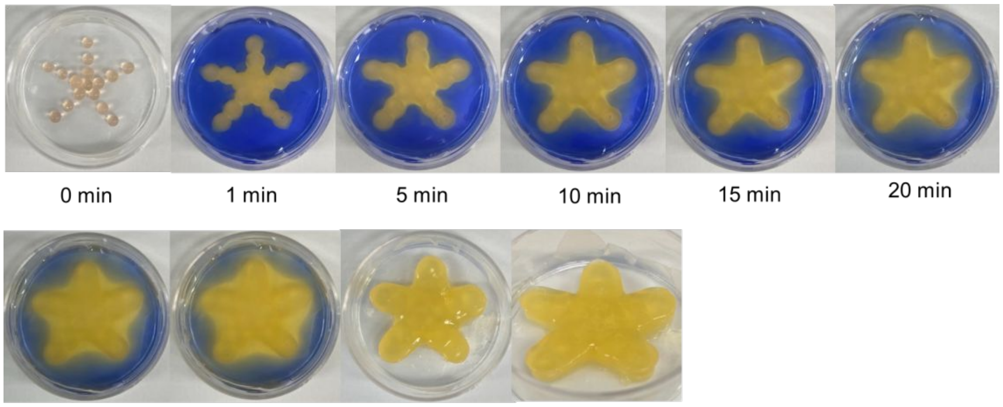

Figure S32 The formation of free-standing hydrogel objects based on five hybrid gel beads loaded with acetic acid (0.062 mmol/bead) in the 3.5 cm petri dish containing the DBS-carboxylate (0.3% wt/vol, 3mL).

S3.3 Imprinting in Diffusion-Adhesion Gel Assembly

Acetic acid-loaded gel bead: Acetic acid (3 mL, 1 M) was prepared, followed by the addition of thymol blue (15 µL, 1% in EtOH). Ten gel beads were then soaked in this solution. Gel beads loaded with acetic acid indicated an orange-pink colour.

DBS-carboxylate-loaded gel beads: DBS-COOH (3 mg) in water (1 mL total) was dissolved by NaOH (60 µL, 0.5 M) then sonicated until complete solution. Thymol blue (9 µL, 1% in EtOH) was added into the solution, resulting in a colour change to blue. Four gel beads were soaked into the DBS-carboxylate solution. Gel beads loaded with DBS-carboxylate indicated a blue colour.

A DBS-carboxylate-loaded gel bead and an acetic acid-loaded gel bead were isolated and placed in direct contact with each other in the DBS-carboxylate solution (0.3% wt/vol, 3 mL). To see the colour change more clearly, the two gel beads were placed on parafilm. Both of them were in contact. Gel beads that were prepared by immersing ten gel beads in water containing thymol blue (15 µL, 1% in EtOH) were used instead of acetic acid-loaded gel beads as the control experiment.

Shaped and patterned gel using DBS-carboxylate loaded gel bead: DBS-carboxylate-loaded gel beads and acetic acid-loaded gel beads were prepared as described above. DBS-carboxylate-loaded gel bead(s) was/were placed in the petri dish containing DBS-carboxylate solution (0.3%wt/vol, 3 mL), and the acid-loaded gel beads were then placed around it/them. After 30 min, DBS-carboxylate-loaded gel bead(s) was/were simply removed using tweezers.

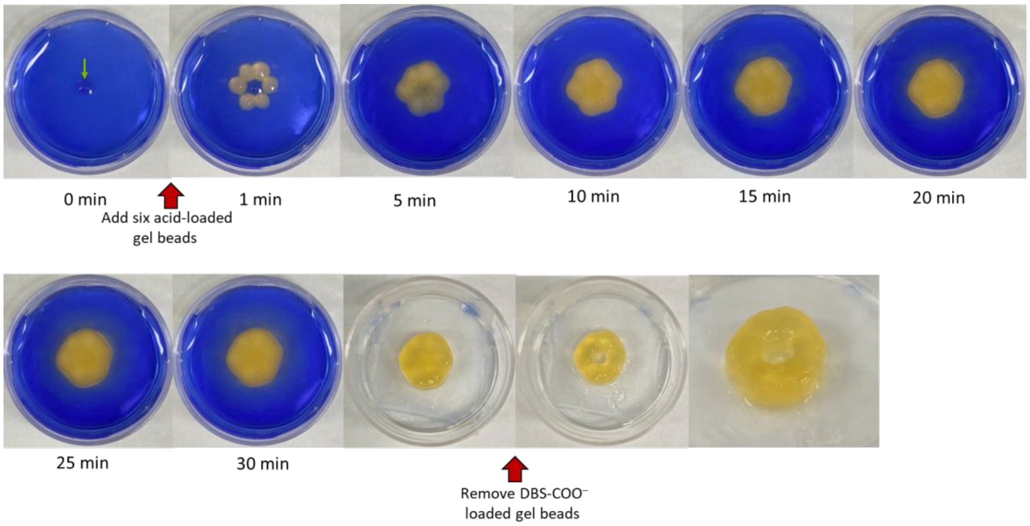

Figure S33 The formation of donut-like shape from the gel beads loaded with acetic acid (0.062 mmol/bead) and DBS-carboxylate in the 3.5 cm petri dish containing the DBS-carboxylate (0.3% wt/vol, 3mL).

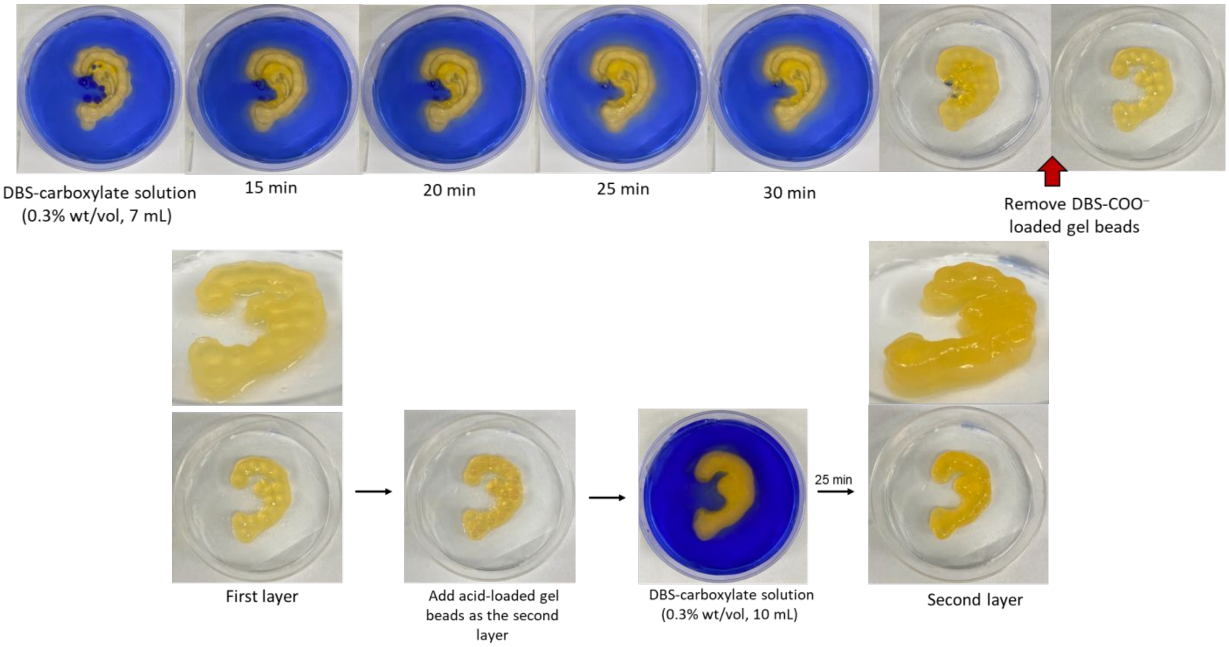

Figure S34 The formation of ear-like shape from the gel beads loaded with acetic acid (0.062 mmol/bead) and DBS-carboxylate (0.3% wt/vol) in the 6.0 cm petri dish containing the DBS-carboxylate (0.3% wt/vol)

S4 Fabrication and characterization of core-shell gel beads incorporating NPs in the core

S4.1 Uptake of $AuCl_3$

Uptake of Au(III) by hydrogels: 1 mL (or 0.5 mL) of each gel prepared (Section 2.1) in water was immersed in 3 mL (or 1.5 mL) of $AuCl_3$ solution (5 mM), respectively. As each gel bead was prepared in a 20 µL volume, 25 gel beads, which were estimated to be equivalent to 0.5 mL gel, were immersed in 1.5 mL of $AuCl_3$ solution (5 mM). After 24 h, aliquots of supernatant were transferred to a cuvette and diluted with water (giving 2 mL total). UV absorbance at 305-310 nm was recorded. This experiment was performed in triplicate for each gel and average values are reported. A colour change from translucent hydrogel to dark purple was observed in the samples, which indicated that Au(III) was reduced to Au(0).

Table S5 $AuCl_3$ uptake in each hydrogel. The concentration is presented in the unit of % wt/vol.

Gel	gel volume	LMWG Loading	Agarose Loading	Total Loading	µmoles Au /mL gel	µmoles DBS-$CONHNH_2$ /mL gel	ratio of Au:DBS-$CONHNH_2$	% Au uptake
DBS-$CONHNH_2$ vial	1 mL	0.3%	-	0.3%	13.2	6.3	2	88
DBS-$CONHNH_2$/Agar vial	1 mL	0.3%	1.0%	1.3%	11.7	6.3	2	78
DBS-$CONHNH_2$/Agar beads	25 beads	0.3%	1.0%	1.3%	14.1	6.3	2	94
DBS-$CONHNH_2$/Agar vial	0.5 mL	0.3%	1.0%	1.3%	14.2	6.3	2	95

TEM of gel bead loaded by $AuCl_3$: AuNP-loaded gel bead cores were prepared as described above. After 24 h, the supernatant was removed, and the gels were washed with water multiple times. Samples

for TEM were obtained by adding a small amount of each sample on a copper grid. The excess of sample was removed with filter paper and allowed to set. A negative stain (1% uranyl acetate) was then added. Before the images were taken, the samples were left to rest for 30 minutes.

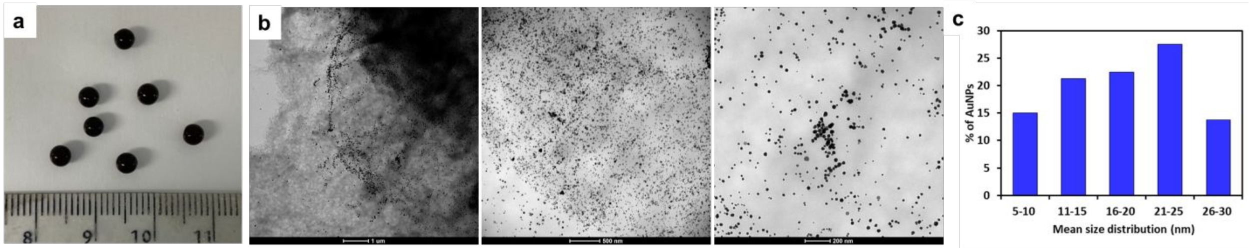

Figure S35 (a) Photograph of AuNPs-loaded gel beads. (b) TEM images of gel bead incorporating AuNPs. Scale bar: 1 μm, 500 and 200 nm (from left to right). (c) Size distribution of diameters of AuNPs incorporated within gel bead

S4.2 Growing symmetric spherical shell on AuNP-loaded gel bead core

AuNP-loaded gel bead cores were prepared as described in Section 3.1, and core-shell gel beads were fabricated as described in Section 2.1 using an AuNP-loaded gel bead as the core.

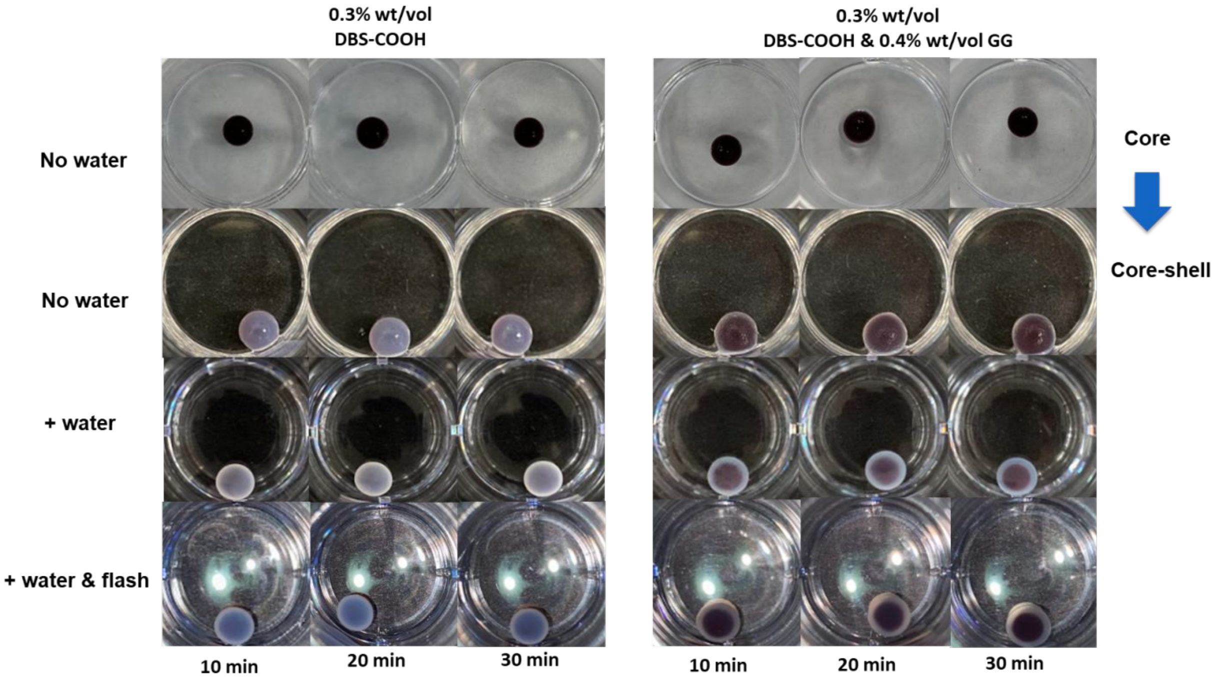

Figure S36 Photographs of DBS-CONHNH$_2$/agarose/DBS-COOH (left) and DBS-CONHNH$_2$/agarose/DBS-COOH/GG (right) core-shell bead which DBS-CONHNH$_2$/agarose core was loaded with AuNPs, compared with the initial DBS-CONHNH$_2$/agarose gel bead core. The gel bead cores were prepared by soaking ten gel beads in acetic acid (1 M, 3 mL, 30 min). The photos of core-shell gel beads in each well were taken in different ways (without water, with water and with water plus flash), to visualise the core-shell structure.

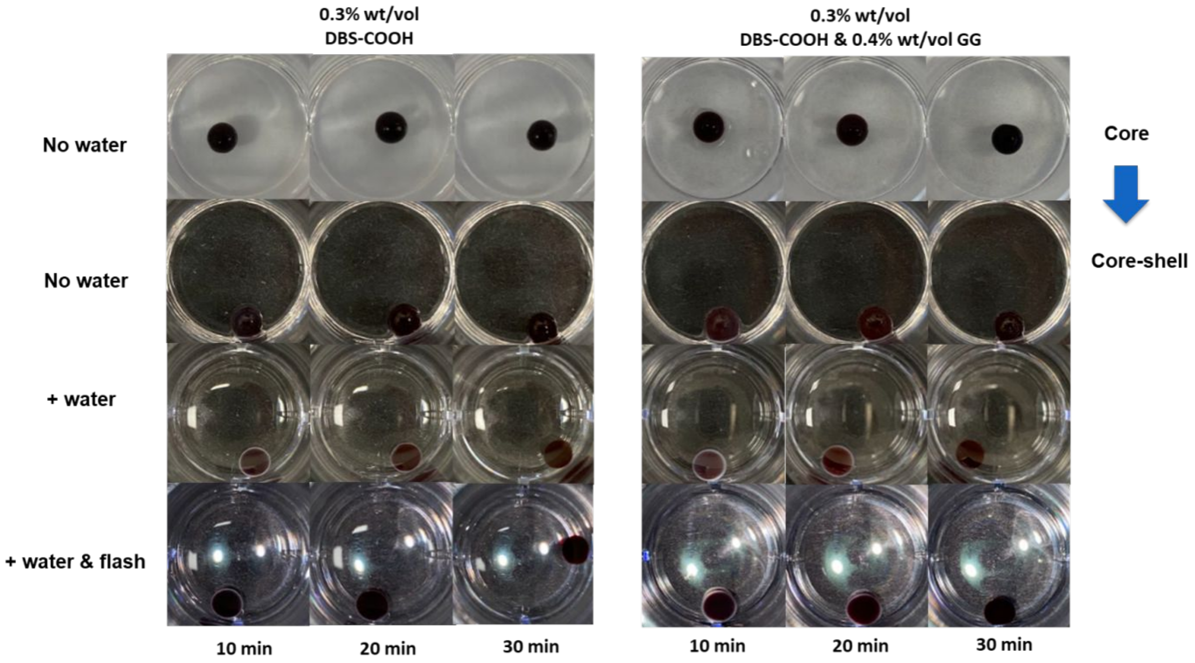

Figure S37 Photographs of DBS-CONHNH$_2$/agarose/DBS-COOH (left) and DBS-CONHNH$_2$/agarose/DBS-COOH/GG (right) core-shell bead which DBS-CONHNH$_2$/agarose core was loaded with AuNPs, compared with the initial DBS-CONHNH$_2$/agarose gel bead core. The gel bead cores were prepared by soaking ten gel beads in acetic acid (0.5 M, 3 mL, 30 min). The photos of core-shell gel beads in each well were taken in different ways (without water, with water and with water plus flash), to visualise the core-shell structure.

S4.3 NMR studies

Quantification of acetic acid loaded in a Au-loaded core gel bead: Samples were prepared as described in Section 2.2, only using gel beads loaded with AuNPs (Section S3.1).

Table S6 Summary of acetic acid loaded per AuNPs-loaded gel bead core.

initial concentration of acetic acid (M), 3 mL	initial amount of acetic acid (mmol)	integral of acetic acid	integral of DMSO (multiplied by 10)	final amount of acetic acid (mmol)	amount of acetic acid (mmol/ bead)
1.00	3.00	609.22	60.00	2.45	0.055
0.50	1.50	328.54	60.00	1.32	0.018
0.25	0.75	156.14	60.00	0.63	0.012
0.10	0.30	66.34	60.00	0.27	0.003

S4.4 Infrared (IR) spectroscopy

Samples for infrared were prepared as described in Sections 2.1, 3.1 and 3.2. The solvent was then removed from the gels under vacuum. A small amount of the resulting powder was analysed by IR spectroscopy.

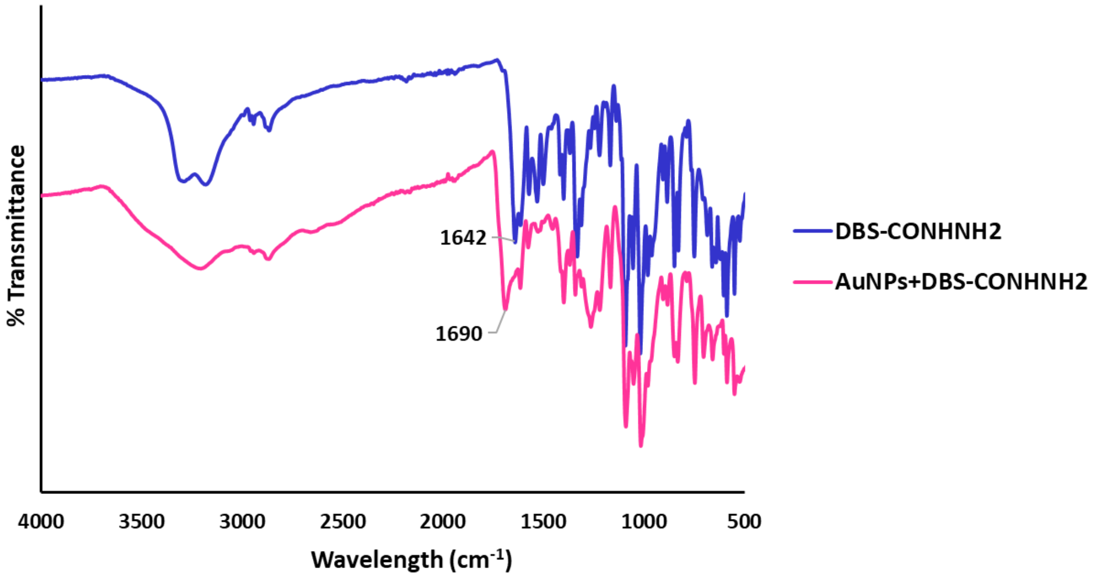

Figure S38 IR spectra of xerogel obtained from DBS-CONHNH$_2$ bulk gel (0.3% wt/vol) with (pink line) and without (blue line) AuNPs.

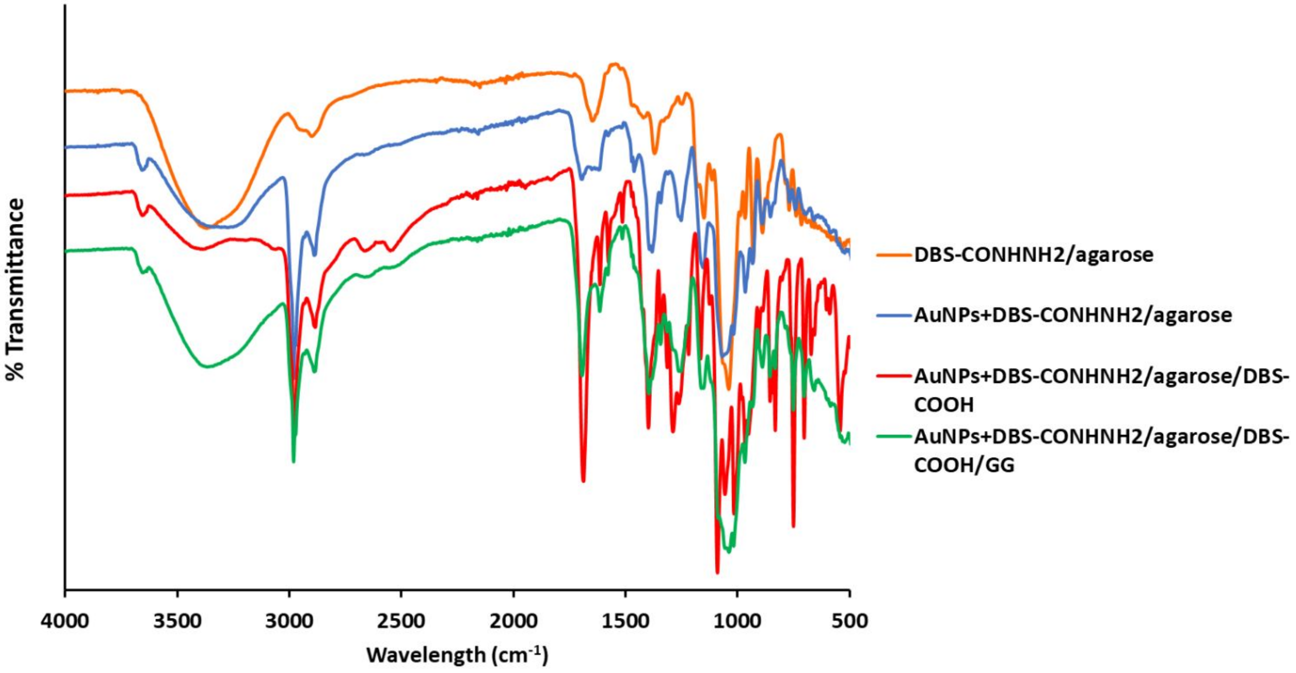

Figure S39 IR spectra of xerogel obtained from DBS-CONHNH$_2$/agarose gel bead (0.3% wt/vol of DBS-CONHNH$_2$ and 1.0% wt/vol of agarose) with (blue line) and without (orange line) AuNPs, DBS-CONHNH$_2$/agarose/DBS-COOH core-shell bead (AuNPs-loaded core, red line) and DBS-CONHNH$_2$/agarose/DBS-COOH/GG core-shell beads (AuNPs-loaded core, green line).

S4.5 Thermal stability studies

All the gels were prepared in 7 ml vials (diameter = 2 cm, height = 6 cm) as described in Sections 2.1 and 2.4, loaded with $AuCl_3$ (5 mM, 1.5 mL for 0.5 mL core gel or 3 mL for 1 mL gel, Section 3.1). T_{gel} measurements were conducted as described in Section 2.4. These experiments were performed in triplicate to ensure reproducibility and the average was reported. Errors are estimated at ±2°C.

Table S7 T_{gel} values of gels loaded with $AuCl_3$. Concentrations are shown in the unit of % wt/vol.

Gel (1 mL total volume)	$DBS-CONHNH_2$ Loading	Agarose Loading	DBS-COOH Loading	GG Loading	$AuCl_3$ Loading	T_{gel}/° C
$DBS-CONHNH_2$	0.3%	-	-	-	5 mM, 3 mL	86
Agarose	-	1.3%	-	-	5 mM, 3 mL	>100
DBS-CONHNH2/Agarose	0.3%	1.0%	-	-	5 mM, 3 mL	>100
DBS-COOH on $DBS-CONHNH_2$/Agarose	0.3%	1.0%	0.3%	-	5 mM, 1.5 mL	shell: 78 core: >100
DBS-COOH/GG on $DBS-CONHNH_2$/Agarose	0.3%	1.0%	0.3%	0.4%	5 mM, 1.5 mL	shell: 89 core: >100

S4.6 Rheology

All the gels were prepared in bottomless vials as described in section S2.1 and 2.4, with the core being loaded with $AuCl_3$ (5 mM, 1.5 mL for 0.5 mL core gel or 3 mL for 1 mL gel, Section 3.1). Measurements were performed as described in Section 2.5.

Table S8 Elastic (G') and viscous (G") moduli of hydrogels.

Gel	$DBS-CONHNH_2$ Loading	Agarose Loading	DBS-COOH Loading	GG Loading	$AuCl_3$ Loading	G'	G"	G'/G" Crossover
$DBS-CONHNH_2$	0.3%	-	-	-	-	939	75	5.1%
$DBS-CONHNH_2$	0.3%	-	-	-	5 mM, 3 mL	984	89	6.3%
Agarose	-	1.3%	-	-	-	7075	341	3.2%
Agarose	-	1.3%	-	-	5 mM, 3 mL	10523	478	8.0%
$DBS-CONHNH_2$/Agarose	0.3%	1.0%	-	-	5 mM, 3 mL	16580	1194	3.2%
DBS-COOH on $DBS-CONHNH_2$/Agarose	0.3%	1.0%	0.3%	-	5 mM, 1.5 mL	10201	541	3.5%
DBS-COOH/GG on $DBS-CONHNH_2$/Agarose	0.3%	1.0%	0.3%	0.4%	5 mM, 1.5 mL	13135	953	3.3%

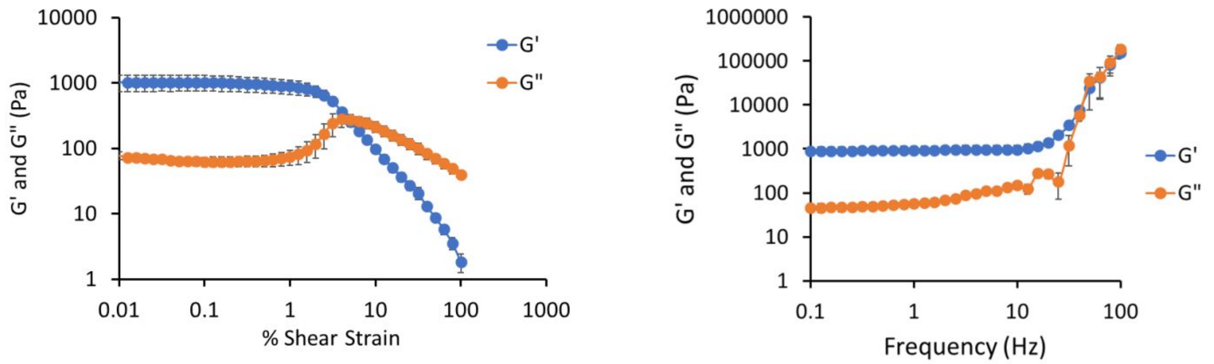

Figure S40 Elastic (G', blue circles) and viscous (G", orange circles) moduli of DBS-CONHNH$_2$ hydrogel (0.3% wt/vol) with increasing shear strain (left) and frequency (right).

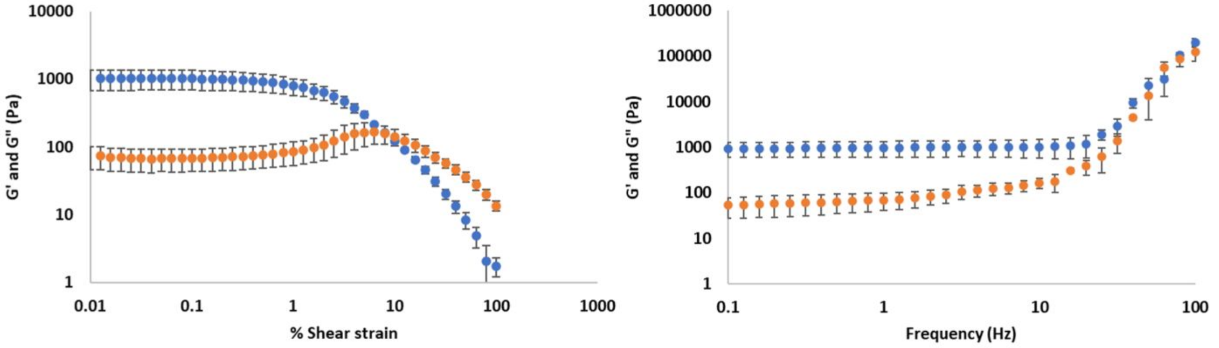

Figure S41 Elastic (G', blue circles) and viscous (G", orange circles) moduli of DBS-CONHNH$_2$ hydrogel (0.3% wt/vol – loaded with $AuCl_3$) with increasing shear strain (left) and frequency (right).

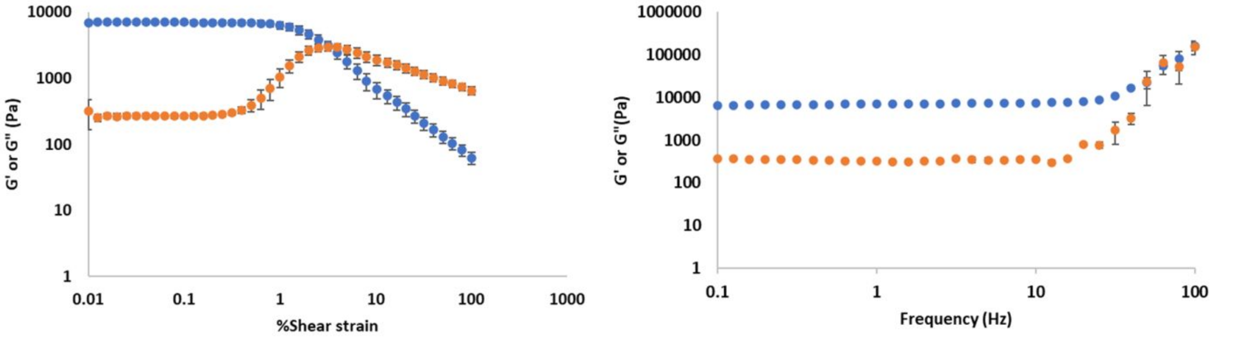

Figure S42 Elastic (G', blue circles) and viscous (G", orange circles) moduli of agarose hydrogel (1.3% wt/vol) with increasing shear strain (left) and frequency (right).

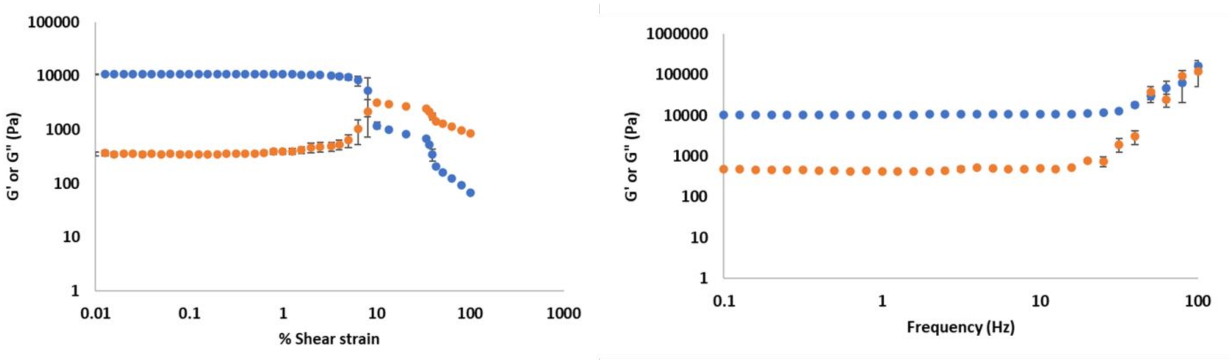

Figure S43 Elastic (G', blue circles) and viscous (G'', orange circles) moduli of agarose hydrogel (1.3% wt/vol – loaded with $AuCl_3$) with increasing shear strain (left) and frequency (right).

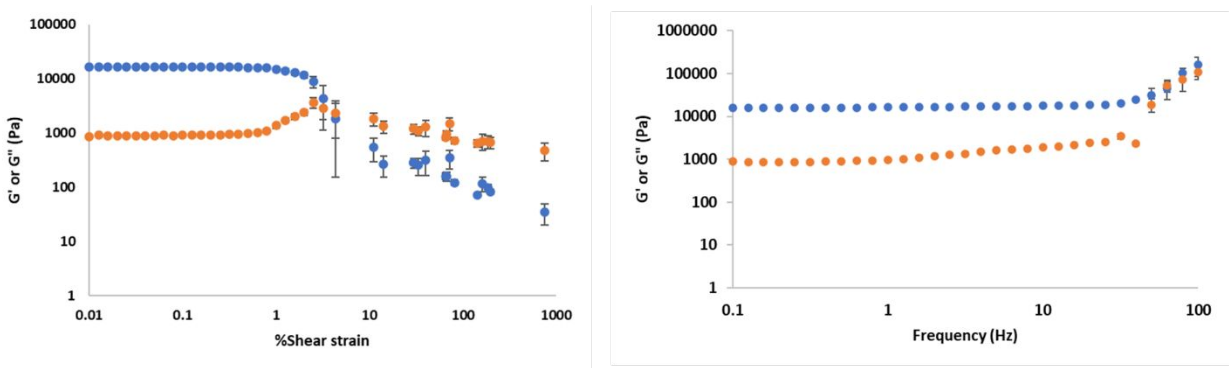

Figure S44 Elastic (G', blue circles) and viscous (G'', orange circles) moduli of DBS-CONHNH$_2$/agarose hydrogel (0.3% wt/vol DBS-CONHNH$_2$ and 1.0% wt/vol agarose– loaded with $AuCl_3$) with increasing shear strain (left) and frequency (right).

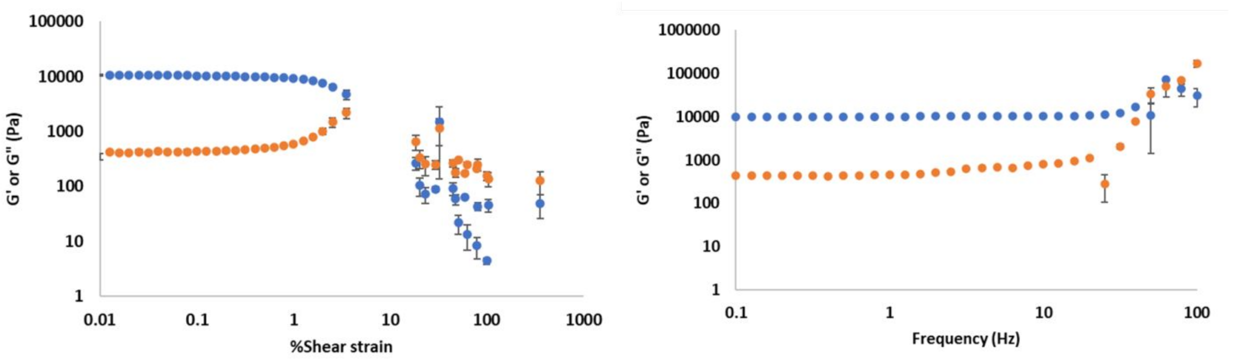

Figure S45 Elastic (G', blue circles) and viscous (G'', orange circles) moduli of core-shell layered DBS-COOH (0.3% wt/vol) on DBS-CONHNH$_2$/agarose hydrogel (0.3% wt/vol DBS-CONHNH$_2$ and 1.0% wt/vol agarose – loaded with $AuCl_3$) with increasing shear strain (left) and frequency (right).

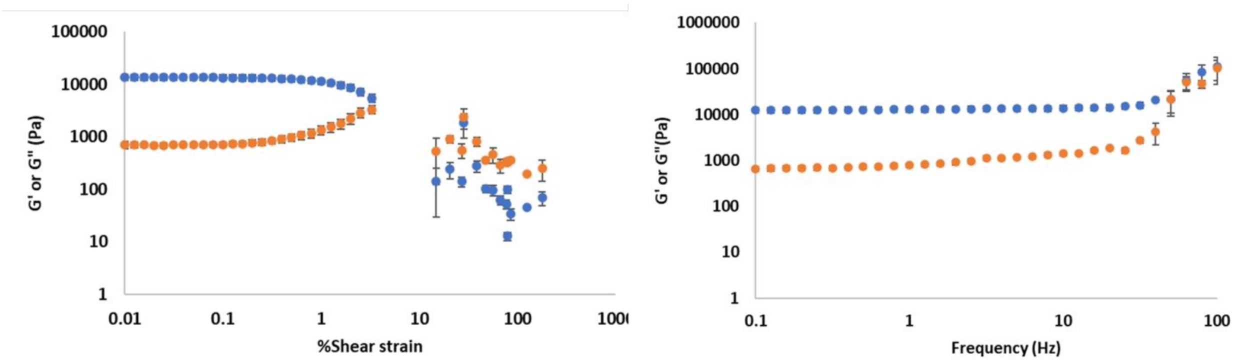

Figure S46 Elastic (G', blue circles) and viscous (G", orange circles) moduli of core-shell layered DBS-COOH/gellan gum (0.3% wt/vol DBS-COOH and 0.4% wt/vol gellan gum) on DBS-CONHNH$_2$/agarose hydrogel (0.3% wt/vol DBS-CONHNH$_2$ and 1.0% wt/vol agarose – loaded with AuCl)$_3$ with increasing shear strain (left) and frequency (right).

S4.7 Optical Microscopy

Core-shell samples for optical imaging were prepared as described in Section 2.1 using AuNP-loaded gel beads (Section 3.1). The images were taken at x30 magnification on the Zeiss Stereo Lumar V12 microscope with Neolumar Sx0.63 objective and images were collected with AxioCam MRc 5 digital camera.

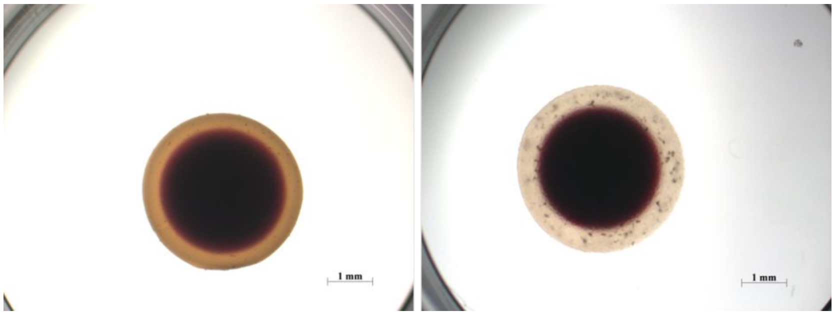

Figure S47 Optical microscopy images of DBS-CONHNH$_2$/agarose/DBS-COOH (left) and DBS-CONHNH$_2$/agarose/DBS-COOH/GG (right) core-shell beads in which the DBS-CONHNH$_2$/agarose core was loaded with AuNPs. Scale bar: 1 mm.

S4.8 Transmission Electron Microscopy (TEM)

Core-shell samples for TEM imaging were prepared as described in Section 3.2. A small amount of shell sample was isolated and placed on a copper grid. The excess sample was then removed with filter paper and the remainder allowed to set. A negative stain (1% uranyl acetate) was then added. Before the images were taken, the samples were left to rest for 30 minutes.

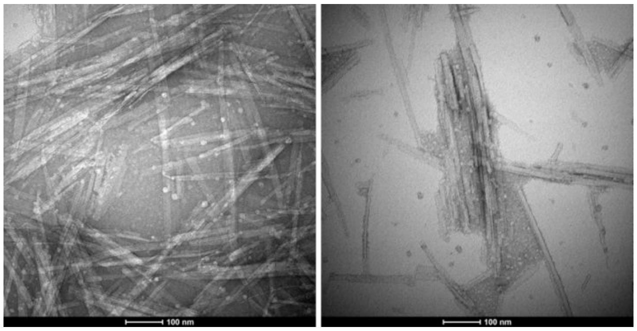

Figure S48 TEM images of DBS-COOH shell from DBS-CONHNH$_2$/agarose/DBS-COOH core-shell bead (DBS-CONHNH$_2$/agarose core incorporating AuNPs). Scale bar: 100 nm.

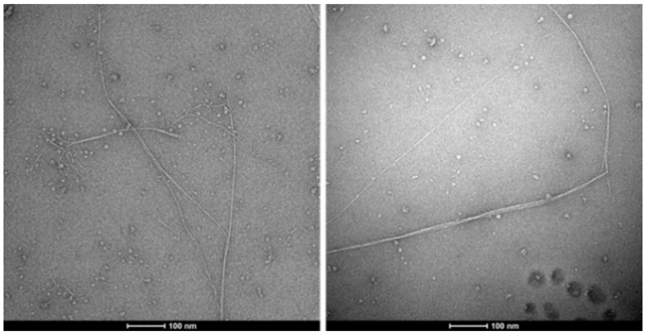

Figure S49 TEM images of DBS-COOH/GG shell from DBS-CONHNH$_2$/agarose/DBS-COOH/GG core-shell bead (DBS-CONHNH$_2$/agarose core incorporating AuNPs). Scale bar: 100 nm.

S4.9 Scanning Electron Microscopy (SEM)

Samples for SEM imaging were prepared as described in Section 3.2. The samples were prepared by freeze drying. Samples were dehydrated by washing with a graded series of acetones then critical point drying by replacing the acetone with liquid CO_2 and subliming the CO_2 to a gaseous state.

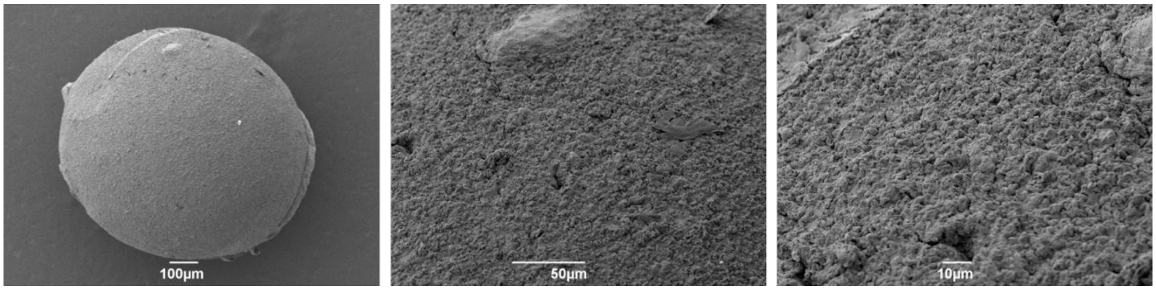

Figure S50 SEM images of DBS-CONHNH$_2$/agarose core gel bead incorporating AuNPs and surface. Scale bar from left to right: 100, 50 and 10 µm.

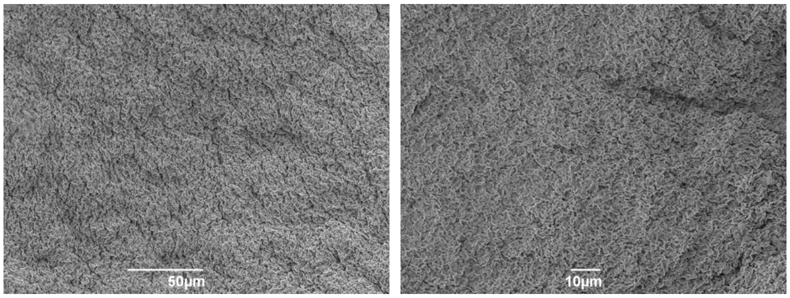

Figure S51 SEM images of DBS-CONHNH$_2$/agarose/DBS-COOH core-shell bead surface (DBS-CONHNH$_2$/agarose core incorporating AuNPs). Scale bar from left to right: 50 and 10 μm.

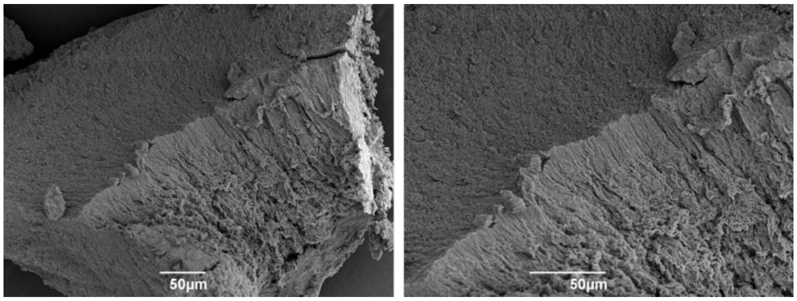

Figure S52 SEM images of shell fracture from DBS-CONHNH$_2$/agarose/DBS-COOH core-shell bead (DBS-CONHNH$_2$/agarose core incorporating AuNPs). Scale bar: 50 μm.

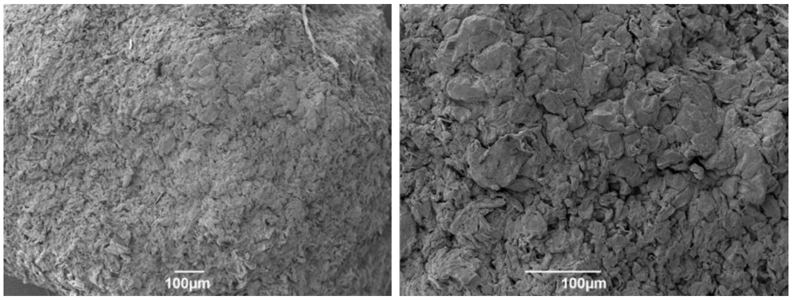

Figure S53 SEM images of DBS-CONHNH$_2$/agarose/DBS-COOH/GG core-shell bead surface (DBS-CONHNH$_2$/agarose core incorporating AuNPs). Scale bar: 100 μm.

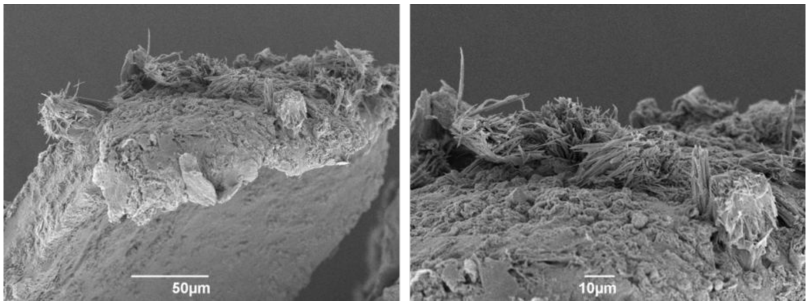

Figure S54 SEM images of shell fracture from DBS-CONHNH$_2$/agarose/DBS-COOH/GG core-shell bead (DBS-CONHNH$_2$/agarose core incorporating AuNPs). Scale bar from left to right: 50 and 10 μm.

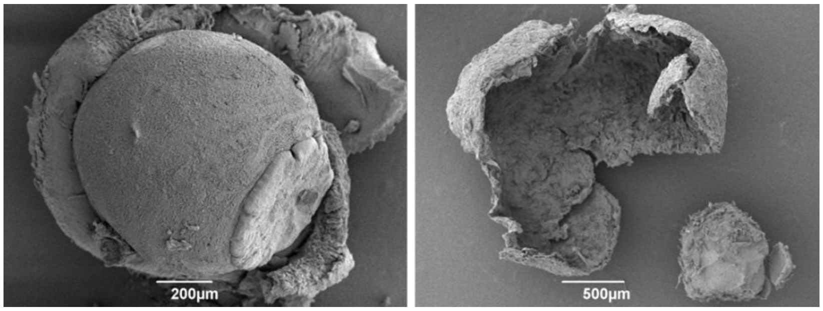

Figure S55 SEM images of DBS-CONHNH$_2$/agarose/DBS-COOH (left) and DBS-CONHNH$_2$/agarose/DBS-COOH /GG (right) core-shell bead (DBS-CONHNH$_2$/agarose core incorporating AuNPs). Scale bar from left to right: 200 and 500 μm.

S4.10 Growing a symmetric spherical shell on a AgNP-loaded core gel bead

Ten DBS-CONHNH$_2$/agarose gel beads (20 μL volume), which were estimated to be equivalent to 0.2 mL of gel, were immersed in 1 mL of $AgNO_3$ solution (10 mM). After 24 h, aliquots of supernatant were removed, and the beads were washed with water multiple times. Ten gel beads were soaked in acetic acid (1 M, 3 mL) for 30 min. Core-shell gel beads were prepared as described in Section 2.1.

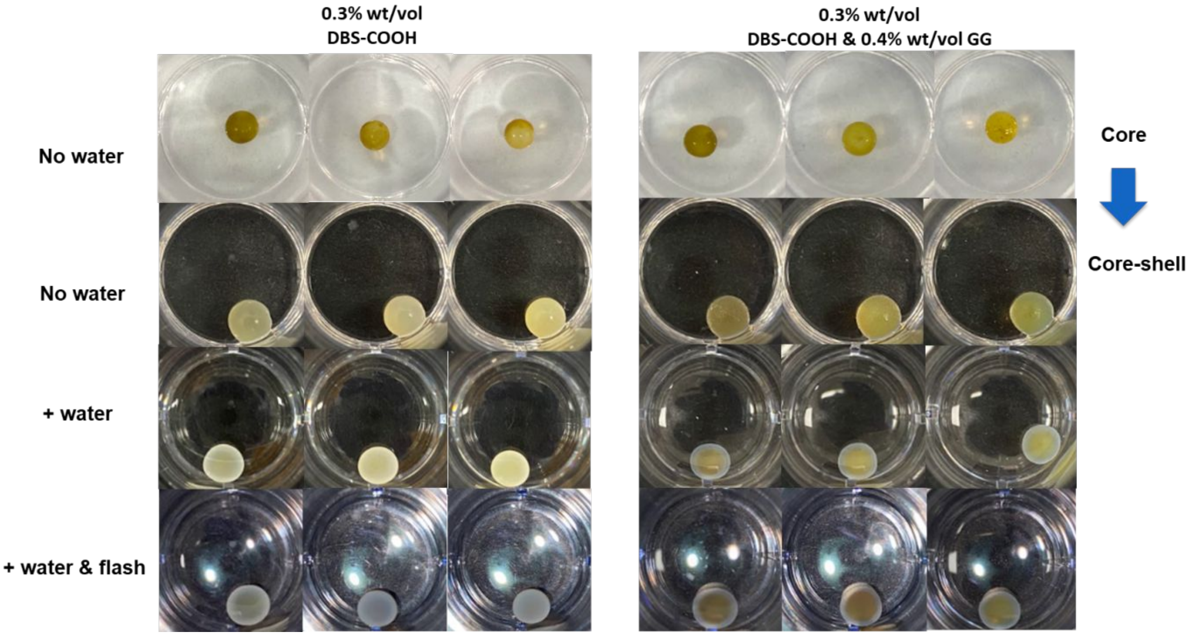

Figure S56 Photographs of DBS-CONHNH$_2$/agarose/DBS-COOH (left) and DBS-CONHNH$_2$/agarose/DBS-COOH/GG (right) core-shell bead, in which the DBS-CONHNH$_2$/agarose core was loaded with AgNPs. The photos of core-shell gel beads in each well were taken in different ways (without water, with water and with water plus flash), to visualise the core-shell structure.

S5 Fabrication of hydrogel objects via diffusion-adhesion with NP-loaded beads

S5.1 Acid diffusion from NP-loaded gel beads

Gel beads incorporating AuNPs or AgNPs were prepared as described in Sections S3.1 and 3.10. Ten NP-loaded gel beads were soaked in acetic acid solution (3 mL, 1 M, 0.5 M, 0.25 M or 0.1 M) for 30 min. DBS-COOH (0.3% wt/vol) in water (5 mL total) was dissolved by addition of NaOH (240 µL, 0.5 M), in the presence of thymol blue (15 µL, 1% in EtOH). Patterned gel objects were prepared by using ten gel beads immersed in acetic acid (1 M) as described in Section 4.2-4.3.

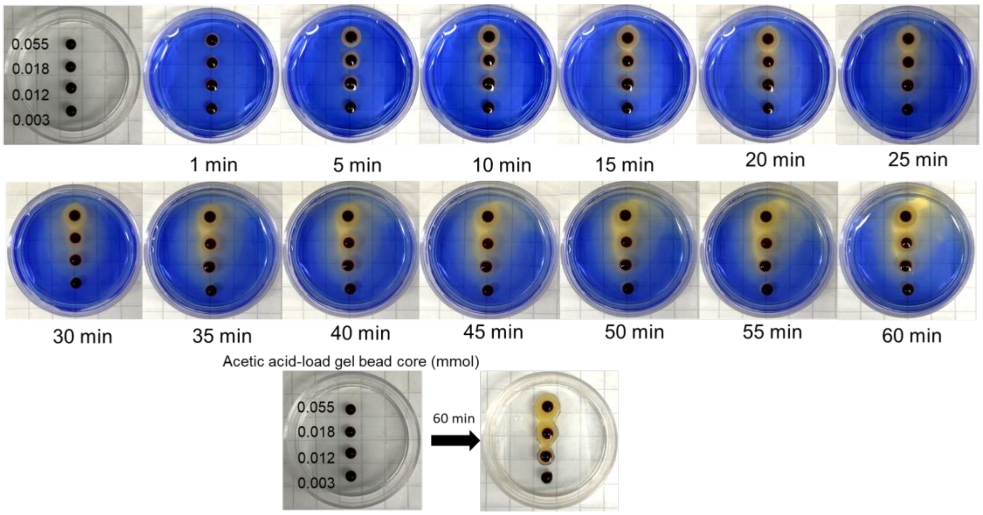

Figure S57 Photographs of growing DBS-COOH gel over time after addition of acetic acid-loaded gel bead into the 3.5 cm petri dish containing the DBS-carboxylate (0.3% wt/vol, 3mL).

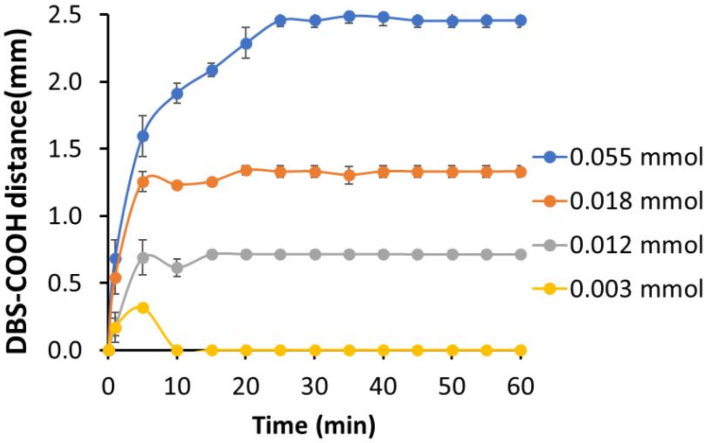

Figure S58 The distance of CH_3COOH diffusion from AuNP-loaded beads as determined by the visualized assembly of radius DBS-COOH shell into an opaque gel over time at different concentrations.

S5.2 Diffusion-adhesion using NP-loaded gel beads

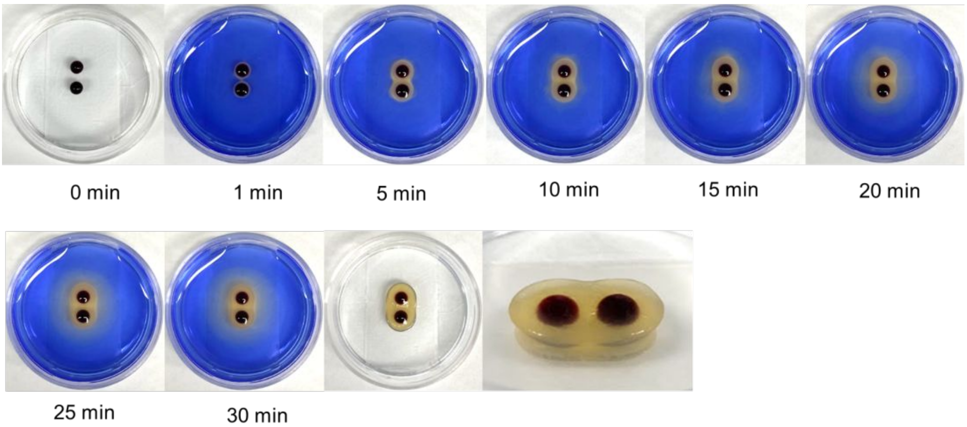

Figure S59 The formation of free-standing hydrogel objects based on two acid-loaded gel beads (0.055 mmol/bead) incorporating AuNPs in the 3.5 cm petri dish containing the DBS-carboxylate (0.3% wt/vol, 3mL).

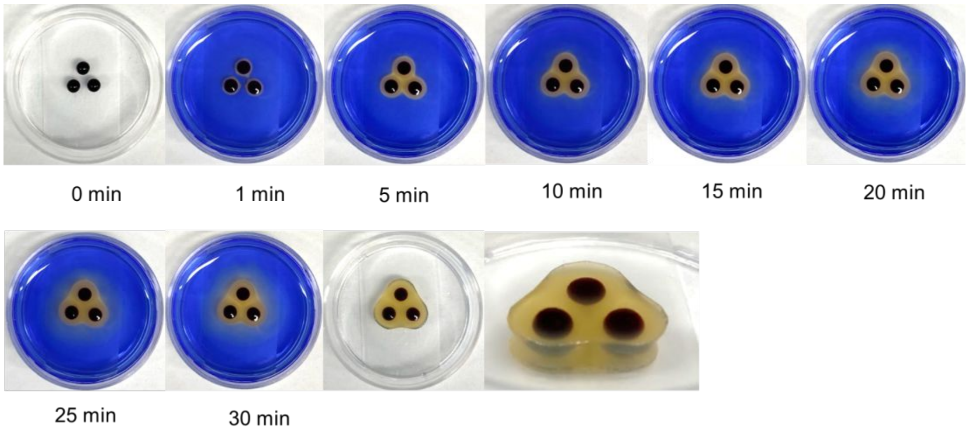

Figure S60 The formation of free-standing hydrogel objects based on three acid-loaded gel beads (0.055 mmol/bead) incorporating AuNPs in the 3.5 cm petri dish containing the DBS-carboxylate (0.3% wt/vol, 3mL).

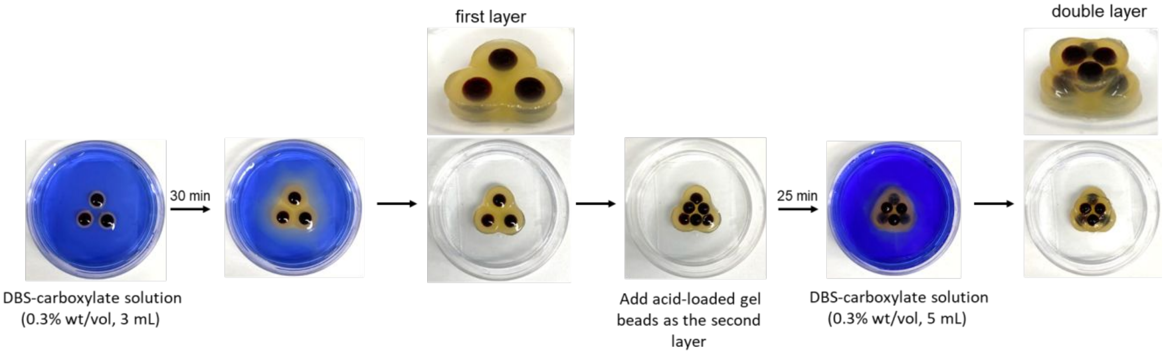

Figure S61 Photograph showing a double triangle fabricated by a layer-by-layer approach from acid-loaded gel beads (0.055 mmol/bead) incorporating AuNPs in the 3.5 cm petri dish containing the DBS-carboxylate (0.3% wt/vol).

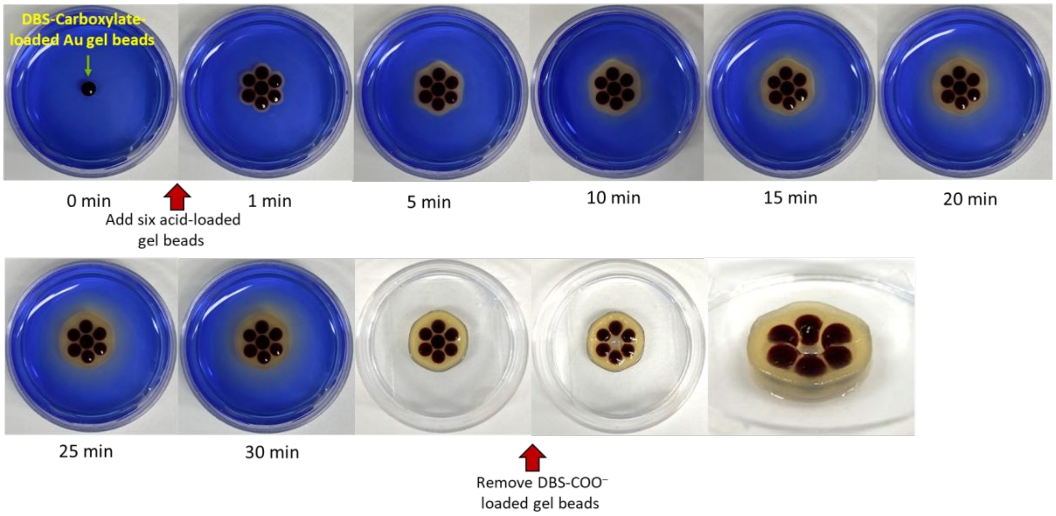

Figure S62 The formation of donut-like shape from acid-loaded gel beads (0.055mmol/bead) incorporating AuNPs in a 3.5 cm petri dish containing DBS-carboxylate (0.3% wt/vol, 3mL).

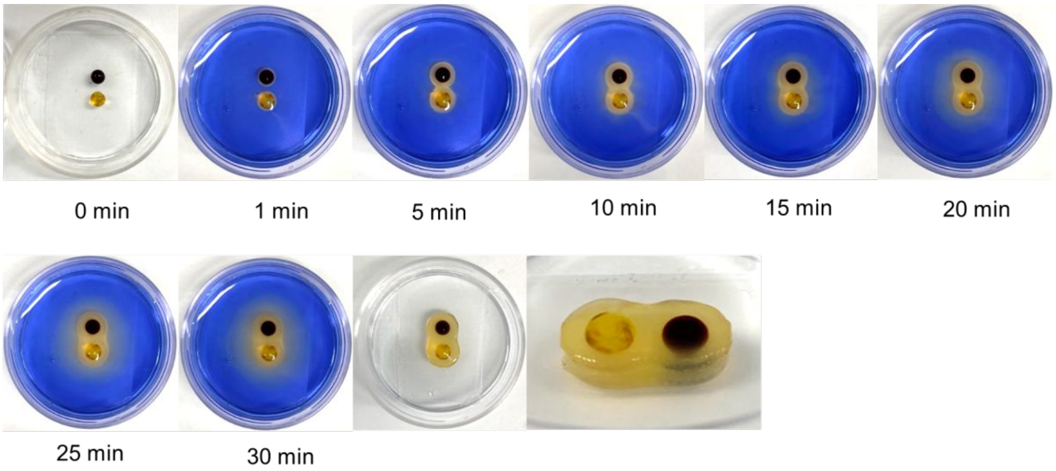

Figure S63 The formation of free-standing hydrogel objects based on two acid-loaded gel beads incorporating AuNPs (dark-purple) or AgNPs (yellow) in a 3.5 cm petri dish containing DBS-carboxylate (0.3% wt/vol, 3mL).

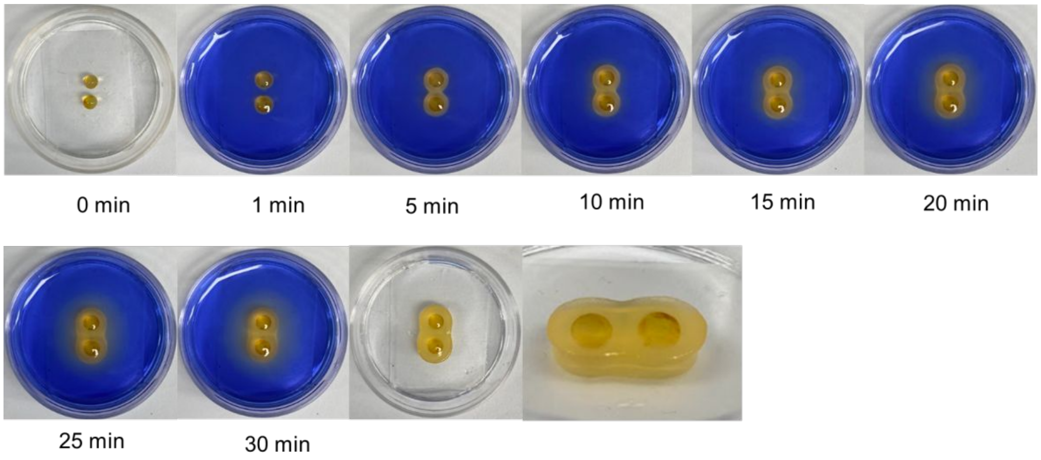

Figure S64 The formation of free-standing hydrogel objects based on two acid-loaded gel beads incorporating AgNPs in a 3.5 cm petri dish containing DBS-carboxylate (0.3% wt/vol, 3mL).

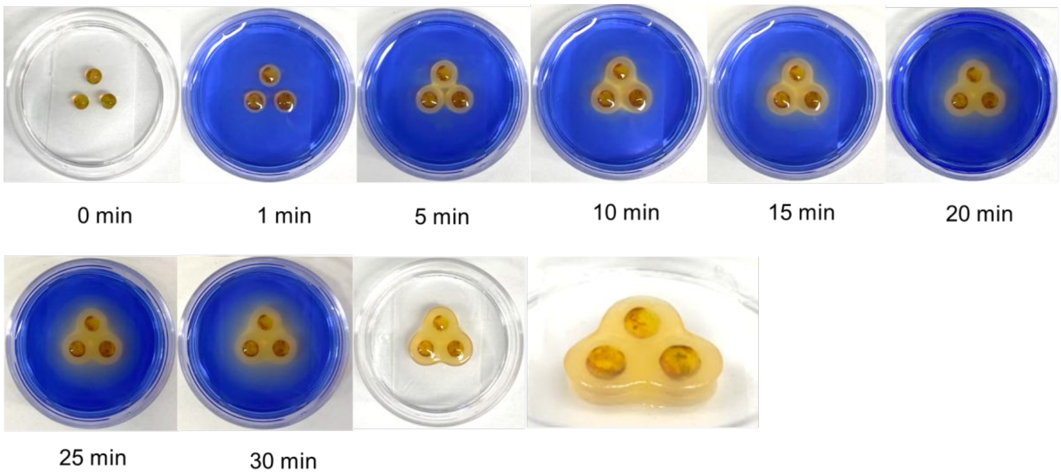

Figure S65 The formation of free-standing hydrogel objects based on three acid-loaded gel beads incorporating AgNPs in a 3.5 cm petri dish containing DBS-carboxylate (0.3% wt/vol, 3mL).

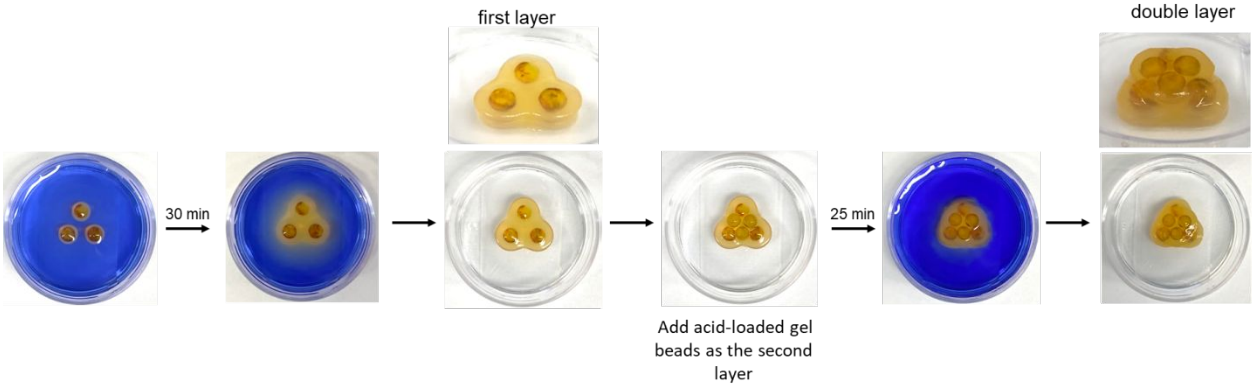

Figure S66 Photographs showing a double triangle fabricated by a layer-by-layer approach from acid-loaded gel beads incorporating AgNPs in a 3.5 cm petri dish containing DBS-carboxylate (0.3% wt/vol).

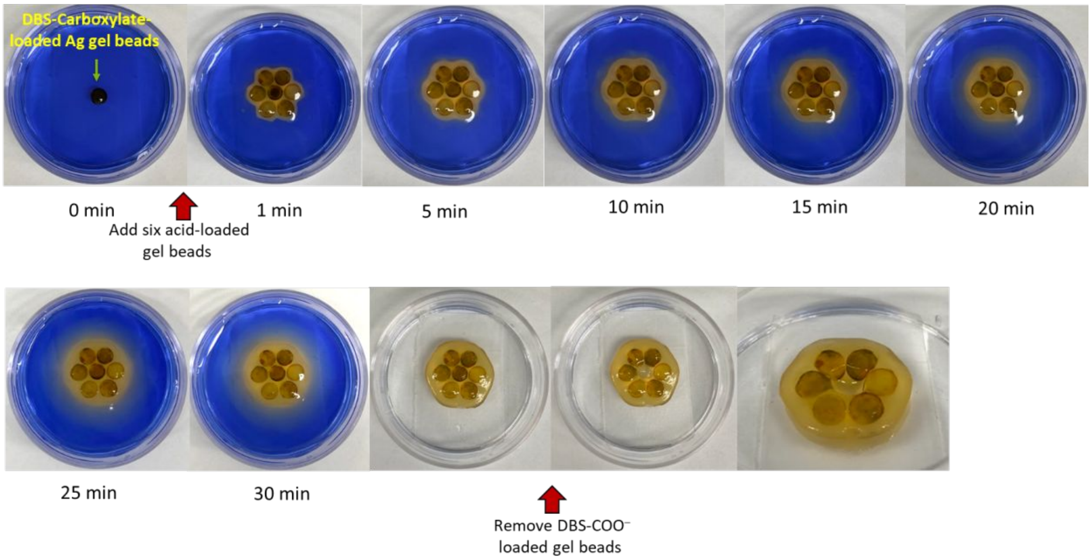

Figure S67 The formation of donut-like shape from acid-loaded gel beads (0.055mmol/bead) incorporating AgNPs in the 3.5 cm petri dish containing the DBS-carboxylate (0.3% wt/vol, 3mL).

S6 References

1. Okesola, B. O.; Smith, D. K. *Chem. Commun.*, **2013**, *49*, 11164-11166.
2. Cornwell, D. J.; Okesola, B. O.; Smith, D. K. *Soft Matter*, **2013**, *9*, 8730-8736.
